# CFTR trafficking mutations disrupt cotranslational protein folding by targeting biosynthetic intermediates

**DOI:** 10.1038/s41467-020-18101-8

**Published:** 2020-08-26

**Authors:** Hideki Shishido, Jae Seok Yoon, Zhongying Yang, William R. Skach

**Affiliations:** 1 CFFT Lab, Cystic Fibrosis Foundation, 44 Hartwell Ave, Lexington, MA 02421 USA; 2 Department of Chemical Physiology and Biochemistry, Oregon Health & Science University, 3181 S.W. Sam Jackson Park Rd, Portland, OR 97239 USA; 3 Cystic Fibrosis Foundation, 4550 Montgomery Ave., Suite 1100N, Bethesda, MD 20814 USA

**Keywords:** Protein folding, Biophysical methods, Protein folding

## Abstract

Protein misfolding causes a wide spectrum of human disease, and therapies that target misfolding are transforming the clinical care of cystic fibrosis. Despite this success, however, very little is known about how disease-causing mutations affect the de novo folding landscape. Here we show that inherited, disease-causing mutations located within the first nucleotide-binding domain (NBD1) of the cystic fibrosis transmembrane conductance regulator (CFTR) have distinct effects on nascent polypeptides. Two of these mutations (A455E and L558S) delay compaction of the nascent NBD1 during a critical window of synthesis. The observed folding defect is highly dependent on nascent chain length as well as its attachment to the ribosome. Moreover, restoration of the NBD1 cotranslational folding defect by second site suppressor mutations also partially restores folding of full-length CFTR. These findings demonstrate that nascent folding intermediates can play an important role in disease pathogenesis and thus provide potential targets for pharmacological correction.

## Abstract (web summary)

Cystic fibrosis (CF) is a lethal genetic disease that is primarily caused by misfolding of the cystic fibrosis transmembrane conductance regulator (CFTR). Here authors show that disease-causing mutations located within the first nucleotide binding domain of CFTR have distinct effects on nascent polypeptides.

## Introduction

Cystic fibrosis (CF) is a lethal genetic disease that is primarily caused by misfolding of the cystic fibrosis transmembrane conductance regulator (CFTR), a polytopic membrane protein with two six-spanning transmembrane domains (TMD1, TMD2), two nucleotide-binding domains (NBD1, NBD2), and a relatively unstructured regulatory (R) domain^1^. Of the >1700 genetic variants reported in the CF gene (www.genet.sickkids.on.ca/cftr), several hundred lead to loss of chloride transport by disrupting CFTR folding and intracellular trafficking^1–3^. The most common disease-associated allele, a three base pair deletion in NBD1 (F508del), is found in ~87% of people with CF where it disrupts CFTR folding by at least two mechanisms: (i) directly inhibiting NBD1 folding efficiency and/or stability^4–6^; and (ii) destabilizing an intramolecular interface between NBD1 and the 4th intracellular loop (ICL4) in TMD2^6–8^. Over the past two decades, high-throughput screening efforts have identified multiple small molecules, often referred to as corrector molecules, with the ability to partially restore normal F508del CFTR folding in cells^9^, and three such molecules have recently received FDA approval as components of combination drugs to treat CF^10–13^. At present, however, the molecular mechanism(s) by which other inherited mutations disrupt CFTR folding and trafficking remain largely unknown.

For CF and other human diseases caused by aberrant protein folding, it has been difficult to experimentally determine precisely when a mutant protein deviates from its native folding pathway. For example, although mutations have been shown to influence folding intermediates immediately after synthesis^14^, little is known about whether misfolding occurs during synthesis itself. This is particularly relevant because the CFTR corrector molecules lumacaftor and presumably tezacaftor, must be present during CFTR synthesis to optimally stimulate CFTR folding in cells, suggesting that they might act on a transient folding intermediate^15^. In addition, protein folding in cells is coupled to translation as the nascent polypeptide emerges from the ribosome exit tunnel^16–26^. De novo folding is therefore influenced by numerous factors^25,26^, including the presence of the adjacent ribosome^18,20,22,25–30^, translation elongation rate^18,19,25,26,31–34^, molecular crowding^35,36^, and interaction with cellular chaperones^25,26,37–42^. Thus, an important question in understanding basic mechanisms of folding disorders is whether disease-causing mutations prevent the initial acquisition of native structure by disrupting the cotranslational folding landscape.

CFTR folding is intrinsically complex and involves insertion of 12 transmembrane helices into the lipid bilayer, individual folding of soluble domains, and assembly of these domains into the mature protein structure. Some of these steps occur cotranslationally during synthesis, whereas others occur posttranslationally. NBD1 folding is a critical step in this process, and the efficiency of NBD1 folding is a limiting step in CFTR biosynthesis^8,43,44^. In addition, NBD1 does not spontaneously refold in vitro^4,5^, suggesting that the de novo protein folding pathway has a critical role in achieving a native structure. Genetic suppressor mutations that improve NBD1 folding efficiency or ICL4-NBD1 interactions can partially restore intracellular trafficking of F508del and certain other CFTR mutants^6–8^, whereas correction of both defects restores folding to near wild-type levels. These results suggest that therapeutic agents, which improve cotranslational folding efficiency, could provide a pharmacological strategy for treating protein folding disorders.

To better understand how CFTR, and proteins in general, fold in cells, we devised a strategy to interrogate nascent polypeptides as they are synthesized on the ribosome^17,18^. Using this system, we previously showed that NBD1 acquires its structure cotranslationally through a distinct series of carefully choreographed folding events. Folding is initiated by rapid compaction of an N-terminal subdomain (CFTR residues #389–491), and then followed by folding of the α-helical subdomain (residues 500–564), and a parallel four-stranded β-sheet that is buried in the core of the domain (residues 568–603). Although synthesis of these subdomains proceeds sequentially, N-terminus to C-terminus, the timing of these folding events is tightly coordinated to ensure that α-helical subdomain collapse is delayed until the β-sheet core is synthesized.

In the current study, we examine how CF disease-causing mutations impact these cotranslational folding events at defined stages of NBD1 synthesis. We show that: (1) numerous NBD1-folding mutations impact nascent polypeptide folding; (2) two mutations (A455E and L558S) in different regions of NBD1 delay cotranslational compaction of folding intermediates during a similar window of synthesis of the β-sheet core; (3) destabilizing effects of the ribosome augment the mutation-induced folding defect; and (4) correction of this transient defect via second site suppressor mutations partially restores trafficking of full-length CFTR in cells. These studies indicate that de novo folding events can impact final folding outcome and provide insight into how disease-causing mutations contribute to protein folding disorders by perturbing intermediate folding states that transiently populate the folding pathway.

## Results

### CF mutations perturb cotranslational folding intermediates

**Fig. 1:**
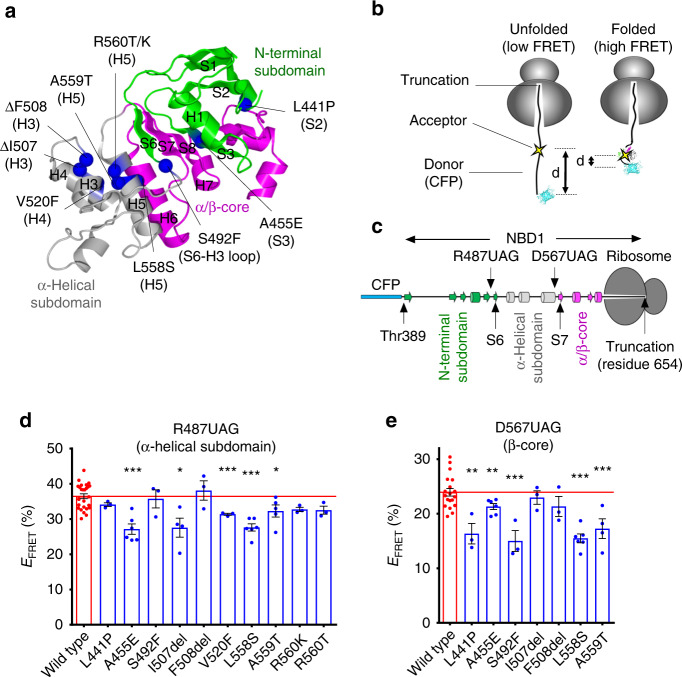
CF-causing mutations perturb NBD1 biosynthetic intermediates. **a** Location of CFTR processing mutations shown in the NBD1 structure taken from the human CFTR Cryo-EM structure (PDB: 5UAK). **b** Cartoon conceptually depicting FRET assay using translationally incorporated fluorophores to detect structural transitions of ribosome-attached nascent polypeptides. **c** Schematic of ribosome-attached CFP-NBD1 fusion protein truncated at CFTR residue 654 showing locations of FRET acceptor dye at CFTR residues Arg487, or Asp567, which report folding of the α-helical subdomain, or β-sheet core, respectively^17,18^. Helices and β-strands are drawn as cylinders and filled arrows, respectively. **d**, **e** FRET efficiencies obtained for wild-type and mutant nascent polypeptides in (ribosome-attached) CFP-NBD1 constructs truncated at residue 654 and containing acceptor dye at residue Arg487 **d** or Asp567 **e**. Each dot represents data from an independent experiment. Each bar shows mean ± SEM, *n* ≥ 3 independent experiments. Two-tailed student’s *t* test comparing wild-type and indicated mutant, asterisk *p* < 0.05, double asterisk *p* < 0.01, triple asterisk *p* < 0.001, n.s. > 0.05 otherwise indicated. Red line shows mean of FRET efficiency for wild-type. Source data of **d** and **e** are provided as a Source Data file.

Numerous missense mutations that disrupt CFTR folding have been identified within CFTR NBD1 subdomains, including the N-terminal subdomain (L441P and A455E), the N-terminal/α-helical subdomain interface (S492F), and the α-helical subdomain (I507del, F508del, V520F, L558S, A559T, R560K, and R560T)^2,45^ (Fig. 1a). To determine whether these mutations influence the cotranslational folding pathway, we used fluorescence resonance energy transfer (FRET) to monitor the overall state of protein compaction as the nascent polypeptide is synthesized on the ribosome (Fig. 1b). This approach involves inserting two fluorescent molecules consisting of an N-terminal donor fluorophore, cyan fluorescent protein (CFP), and an internal small acceptor dye (7-nitrobenz-2-oxa-1,3-diazole) into the nascent polypeptide in 1:1 stoichiometry during synthesis^46–48^ (Fig. 1b, c & Supplementary Fig. 1a–c). Because protein folding principally involves compaction of an extended polypeptide, many residues that are distant in primary structure are brought into close proximity as native structure is acquired^48^. For appropriate donor and acceptor probe sites, an increase in FRET efficiency therefore provides a measure of compaction as the nascent polypeptide grows in length and transitions from an elongated random (unfolded) conformation to a compacted (folded) conformation^17,18,49^.

To examine NBD1 nascent folding intermediates, in vitro-transcribed RNA was truncated in the NBD1-coding sequence and expressed in a rabbit reticulocyte lysate (RRL) cell-free translation system. The terminal stop codon was removed by RNA truncation, which stably binds the nascent polypeptide via a peptidyl-tRNA bond to tRNA at the ribosome P-site, thus generating a uniform cohort of nascent ribosome-attached polypeptides that can be isolated and analyzed^17,18,46,47^ (Supplementary Fig. 1a–c). We previously showed that when nascent NBD1 is synthesized in this system, it folds into a native-like structure that is capable of binding ATP^17^. We also identified several permissive sites for acceptor dye incorporation that do not grossly perturb NBD1 folding^17,18^. The ability to monitor NBD1 folding while attached to the ribosome thus allows access to native folding intermediates that exist only transiently during CFTR synthesis. By translating a series of RNA transcripts truncated at defined lengths, it is possible to capture kinetically stalled translation intermediates that reflect snapshots of the folding process and thus to reconstruct the relative compaction of different subdomains as a function of nascent polypeptide length^17,18,47,48,50,51^. Moreover, RRL mimics the folding environment of the cell in that it includes a broad range of cellular chaperones^52^ (e.g., HSP40, HSP70, HSP90, etc.), degradation machinery (ubiquitin proteasome pathway)^53^, and molecular crowding (~20% cytosol ~80 mg/ml protein). An important caveat is that truncated polypeptides may lack critical native stabilizing contacts and therefore represent a structural ensemble rather than a predominant stable structural intermediate. Truncated cohorts are therefore referred to simply as nascent polypeptides or folding intermediates. Structural perturbations detected by changes in FRET may thus reflect changes in the ensemble that ultimately gives rise to the final folded structure.

Initial studies were carried out using nascent polypeptides truncated at CFTR residue 654 with the acceptor dye inserted either at residue Arg487 (near the S6 β-strand) or Asp567 (near the S7 β-strand), which report on folding of the α-helical subdomain, and the β-sheet core, respectively^18^ (Fig. 1c, Supplementary Fig. 1a). This truncation represents a critical intermediate in which the β-sheet core is in the process of acquiring its native structure^18^, which in turn is temporally coupled to α-helical subdomain compaction^18^. Mutations that alter the timing of α-helical subdomain compaction for this intermediate state would therefore most likely negatively impact CFTR folding. As shown in Fig. 1d, several, but not all trafficking mutations decreased α-helical subdomain compaction as measured by small but significant decreases in FRET efficiency for nascent chains containing the acceptor dye at Arg487. An overlapping set of mutations, L441P, A455E, S492F, L558S, and A559T, but not I507del or F508del, decreased compaction of the β-sheet core (Fig. 1e). Thus, multiple trafficking mutations can cotranslationally perturb NBD1-folding intermediates. As NBD1 folding involves a complex series of steps, it is perhaps not surprising that some mutations do not affect the cotranslational pathway, but exert their effect elsewhere. As we showed previously, F508del had little effect on FRET using this assay^18^, which could indicate either that, (i) it does not affect NBD1 translation intermediates per se, (ii) changes were not detected by these specific probe sites, or (iii) changes were masked by the reduced translation temperature (24 °C), which is known to suppress the folding defect.

### A455E and L558S transiently alter NBD1 folding pathway

**Fig. 2:**
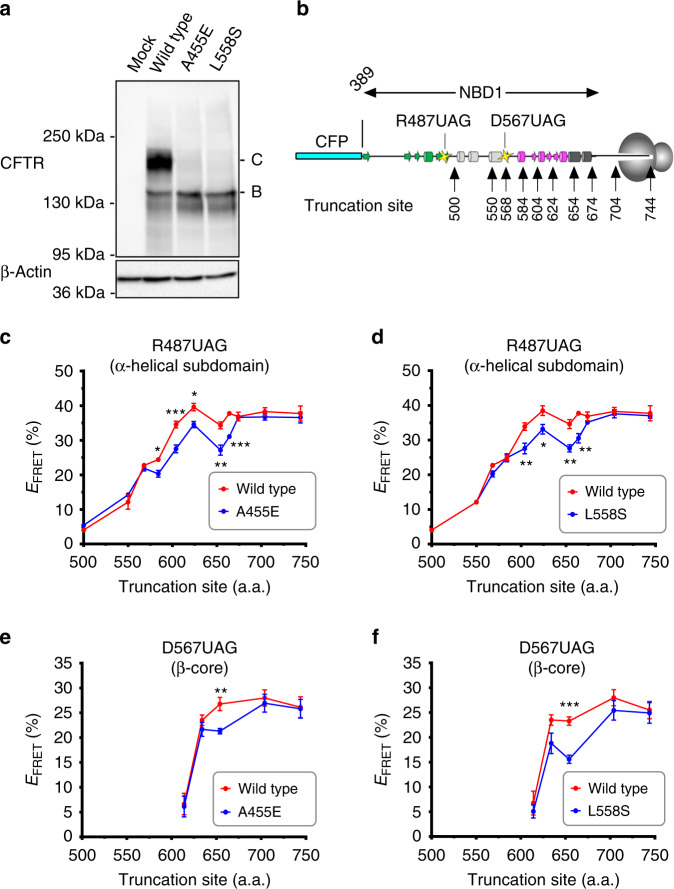
A455E, L558S transiently disrupt NBD1 cotranslational folding. **a** Immunoblot of full-length CFTR (wild-type, A455E, or L558S) expressed in human embryonic kidney (HEK) 293 cells showing core glycosylated (band B) and mature CFTR (band C). Uncropped blots in Source Data. **b** Schematic of ribosome-attached CFP-NBD1 showing approximate location of acceptor dye incorporation sites (residue Arg487 or Asp567) and truncation sites used in **c**–**f**. **c**–**f** Length-dependent FRET efficiencies for wild-type (red line) and A455E or L558S (blue line) obtained from ribosome-attached CFP-NBD1 constructs with acceptor dye located at R487UAG **c**, **d** or D567UAG **e**, **f** for each truncation site indicated. Data are mean ± SEM, *n* ≥ 3 independent experiments. Two-tailed student’s *t* test comparing wild-type and A455E or L558S, asterisk *p* < 0.05, double asterisk *p* < 0.01, triple asterisk *p* < 0.001, n.s. > 0.05 otherwise indicated. Note that wild-type data for truncation sites 500, 568, 584, and 664, and A455E data for truncation sites 500, 550, 568, 664 shown in **c**, and wild-type data for truncation sites 500, 550, 568, 584, and 664, and L558S data for truncation site 664 shown in **d** have error bars smaller than the dots. Some data for truncation sites 568, 604, 654, 666, 704, and 744 shown in **c**, **d** are also taken from Figs. 1d, 3a, b, 4a, b, & 5b. Source data of **a**, **c**–**f** are provided as a Source Data file.

Three mutations, A455E, L558S, and A559T, demonstrated statistically significant folding disruptions of both α-helical subdomain and β-sheet core (Fig. 1d, e). As L558S showed a larger change in FRET than A559T, we focused subsequent studies on A455E and L558S, located within the N-terminal and α-helical subdomains, respectively. Both of these mutations induce a severe block in CFTR trafficking to the cell surface (Fig. 2a), consistent with previous studies^2^. We next tested the effects of A455E and L558S on nascent chains of increasing length generated from sequentially truncated RNA transcripts (Fig. 2b). Wild-type nascent polypeptides with an acceptor dye located at Arg487 underwent a gradual compaction of the α-helical subdomain during synthesis of residue 500 to residue 624 (FRET efficiency increase from 4.1 ± 0.8% to 39.6 ± 1.1% (mean ± SEM, *n* ≥ 3 independent experiments), respectively (Fig. 2c, d), as previously reported^18^. For the A455E mutant, a decrease in FRET (4.1 ± 1% decrease) from that of wild-type was first observed at truncation 584, suggesting that compaction of these nascent polypeptides deviates from wild-type ~100 residues after the mutant residue is synthesized (Fig. 2c). In contrast, the effect of L558S (located in H5) on the α-helical subdomain was first observed at truncation 604, almost immediately after the mutant residue entered the cytosol (6.3 ± 2.0% decrease) (Fig. 2d). The most profound effect of both mutations on the α-helical subdomain compaction was observed at truncation 654 (7.2 ± 0.9% decrease for A455E and 7.0 ± 1.0% decrease for L558S). In addition, FRET efficiency approached wild-type levels at truncation residue 674 when NBD1 synthesis was completed^18^. Note that 30–40 residues of the nascent polypeptide are sequestered within the ribosome exit tunnel. Tertiary folding is therefore limited to cytosolically exposed residues that are similarly offset from the truncation site, and H5 emerges from the ribosome during synthesis of residues 584–604. Both A455E and L558S therefore disrupt NBD1-folding intermediates during a very specific window of synthesis as α-helix H5 to β-strand S7 emerge from the ribosome. Because H5 is the location of numerous CFTR-trafficking mutations as well as second site suppressor mutations that partially correct F508del CFTR folding^8^, this region of NBD1 likely makes critical contacts during initial collapse and compaction of the NBD1 core.

In contrast to the α-helical subdomain, compaction of the wild-type β-sheet core occurred more abruptly and at a slightly later stage of synthesis (between truncations 614–654) (Fig. 2e, f). Both A455E and L558S also disrupted β-sheet core folding, again, during a similar and relatively narrow window of synthesis that overlapped with effects on the α-helical subdomain (Fig. 2e, f). L558S showed a more prolonged effect and greater change in FRET, suggesting that the mutations alter β-sheet core folding by somewhat different mechanisms.

Results from Fig. 2 indicate that despite being separated by ~100 residues in primary sequence, A455E and L558S alter the NBD1 folding landscape during the same period of translation and thus likely act on similar folding intermediate(s). The effect on FRET also diminished as NBD1 synthesis was extended to residue 674. These findings suggest that both mutations disrupt the coordinated compaction of α-helical, and β-sheet core subdomains. Moreover, the NBD1-folding intermediates appear particularly susceptible to perturbation early during synthesis, but less so as the structure of the domain is stabilized by addition of C-terminal residues. We also analyzed two CFTR variants as negative controls to confirm that results in Fig. 2 are not simply a result of fluctuation of the assay system. M470V is a polymorphism in the N-terminal subdomain that is not associated with disease, whereas G551D, also located in H5, causes CF by disrupting CFTR channel gating but has no effect on folding or trafficking. Neither of these variants produced detectable changes in FRET for any of the critical folding intermediates (truncations 604, 614, and 654, Supplementary Fig. 2a, b), supporting our conclusions that the observed compaction delay is specific for misfolding mutations.

### The ribosome accentuates transient folding defects

We previously demonstrated that the ribosome exerts a destabilizing effect on the nascent polypeptide that transiently delays folding of the α-helical subdomain, thereby keeping it in a more open conformation during synthesis of the β-sheet core^18^. Moreover, this delay was required for proper folding and packing of the β-sheet core as the polypeptide emerges from the ribosome^18^. The current study demonstrates that A455E and L558S further delay folding of both the α-helical subdomain and the β-sheet core and thus alter the normal temporal coupling of these folding events (Fig. 2c–f). We therefore wondered how the tethered ribosome influenced the destabilizing effect of these mutations on nascent polypeptide conformation.

**Fig. 3:**
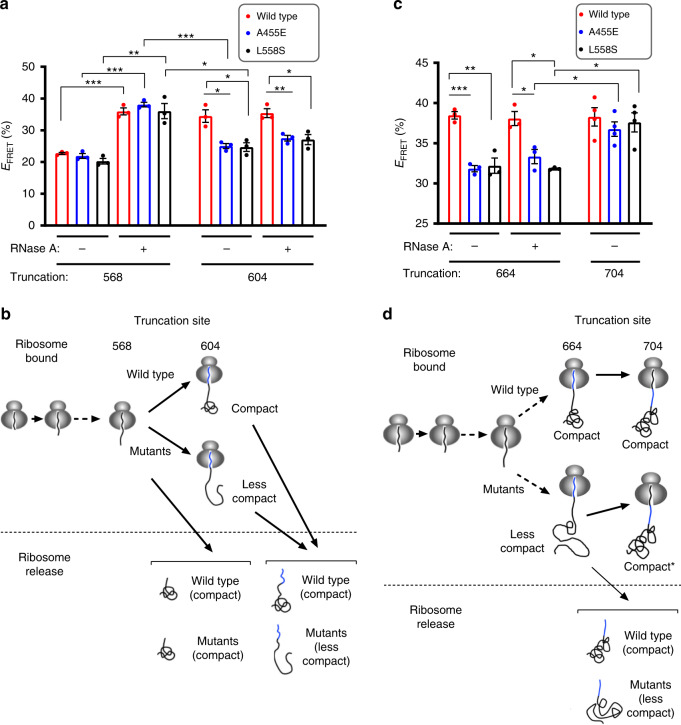
Ribosome influences cotranslational folding defects. **a**, **c** FRET efficiencies of ribosome-bound and released CFP-NBD1 ± A455E or ± L558S polypeptides (acceptor dye at Arg487) truncated at residue 568 or 604 **a**, and 664 or 704 **c**. Each dot represents data from an independent experiment. Each bar shows mean ± SEM, *n* = 3 or 4 independent experiments. Two-tailed student’s *t* test, asterisk *p* < 0.05, double asterisk *p* < 0.01, triple asterisk *p* < 0.001, n.s. > 0.05 otherwise indicated. Ribosome-released (RNase A) (truncation 568 or 664) and ribosome-bound (truncation 604 or 704) polypeptides contains equivalent cytosolically exposed residues, therefore differences in FRET are due to ribosome attachment. **b**, **d** Illustration depicting results of **a**, or **c** showing schematic of wild-type and mutant ribosome-bound and released polypeptides (acceptor dye at Arg487) truncated at indicated residue. **b** NBD1 polypeptides truncated at residue 568 remain unfolded on the ribosome and show an increase in FRET, consistent with α-helical subdomain folding, following ribosome release. Wild-type ribosome-bound and released polypeptides are in the compact sate (high FRET) at truncation 604, whereas mutant polypeptides remain less compact (low FRET) both before and after ribosome release. **d** Wild-type ribosome-bound and released polypeptides are shown in the compact sate at truncation 664, whereas mutant polypeptides remain less compact (low FRET) both before and after ribosome release. In contrast, both wild-type and mutant polypeptides truncated at residue 704 have achieved a compact state (high FRET). Source data of **a** and **b** are provided as a Source Data file.

Figure 3a shows that wild-type and both mutant polypeptides truncated at residue 568 exist in a relatively unfolded state while attached to the ribosome, and all three polypeptides exhibited a substantial increase in FRET following ribosome release. When nascent polypeptides were elongated to truncation 604, the wild-type polypeptide folded on the ribosome and remained folded following release. In contrast, A455E and L558S mutants remained in a more open conformation under both conditions. This finding is consistent with data from Fig. 2 that both mutations exert a disruptive effect on the native conformation at this chain length.

To specifically determine the effect of the ribosome on A455E- and L558S-induced misfolding, we compared FRET obtained from ribosome-released polypeptides truncated at residue 568 to ribosome-attached polypeptides truncated at residue 604 because at these truncations, equivalent residues are cytosolically exposed and available for tertiary folding. As shown in Fig. 3a, mutant polypeptides were less compact than wild-type polypeptides in the ribosome-attached state. Yet, all three peptides (at truncation 568) yielded the equivalent high FRET values after ribosome release, indicating that ribosome attachment appears to augment this particular mutation-induced folding defect (Fig. 3a, b). In other words, the more open conformation observed for mutants at truncation 604 is accentuated by the destabilizing effect that the ribosome exerts on the nascent polypeptide. Moreover, as polypeptides were elongated to residue 604, the mutant polypeptides failed to fold normally even after they were released from ribosome (Fig. 3a, b). This suggests that the nascent (attached) polypeptide has been driven into an off-pathway intermediate by the combined disruptive effects of the mutations and the ribosome, resulting in the inability to fold properly if it is released free into solution at this stage of synthesis.

The second key finding in Fig. 2c, d is that as NBD1 synthesis was completed, mutant nascent polypeptides were eventually able to acquire a compact state, but at a longer truncation length than the wild-type protein. Thus, the net effect of the mutations is to further delay α-helical compaction beyond the normal folding window. We therefore examined the role of the ribosome in mediating this late-stage folding event.

At truncation 664, ribosome-bound A455E and L558S polypeptides still exhibited a less compacted state than wild-type polypeptides (FRET efficiency = 31.8 ± 0.4% for A455E and 32.2 ± 1.0% for L558S), and both mutants failed to fold after ribosome release (Fig. 3c). At truncation 704, however, ribosome-attached wild-type and mutant NBD1 nascent polypeptides yielded equivalent high FRET values (FRET efficiency = 38.3 ± 1.1% for wild-type, 36.8 ± 0.9% for A455E, and 37.6 ± 1.2% for L558S) (Fig. 3c). Because polypeptides released at truncation 664 have equivalent aqueous exposed residues as the attached polypeptides at truncation 704 (Fig. 3d), ribosome attachment must somehow facilitate eventual compaction of the full-length domain. This result is analogous to the previously reported ribosome effect in wild-type polypeptides^18^, but occurs at a longer chain length for the mutant polypeptides. Taken together, our results indicate that ribosome exerts at least two unexpected effects on the mutant nascent polypeptides. It participates in destabilizing the misfolded state at an early stage of synthesis by delaying compaction of the α-helical subdomain and β-sheet core, and subsequently maintains the growing nascent polypeptide in a non-native, but permissive conformation that allows delayed compaction as additional folding information is provided during completion of synthesis (Fig. 3).

### Thermal stability of mutant NBD1-folding intermediates

**Fig. 4:**
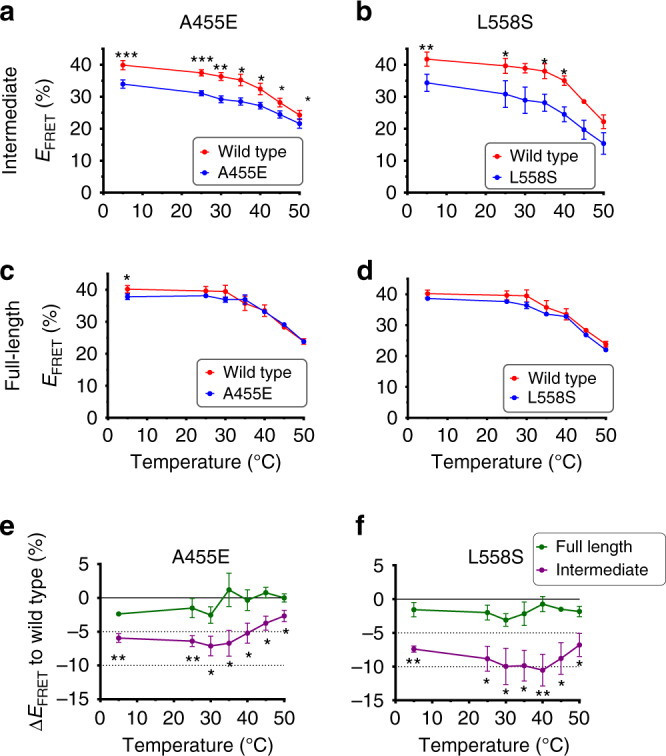
Thermal stability of NBD1-folding intermediates. **a**-**d** Temperature-dependent FRET efficiencies of wild-type (red line) and A455E or L558S (blue lines) ribosome-bound CFP-NBD1 polypeptides. Acceptor dye was located at Arg487 and polypeptides were truncated at residue 654 (intermediate) **a**, **b** or 744 (full-length) **c**, **d**. Data are mean ± SEM, *n* = 3 or 7 independent experiments. Two-tailed student’s *t* test, asterisk *p* < 0.05, double asterisk *p* < 0.01, triple asterisk *p* < 0.001, n.s. > 0.05 otherwise indicated. **e**, **f** Graphs show the difference between temperature-dependent FRET efficiencies for wild-type and mutants calculated from **a**–**d**. One-tailed student’s *t* test, *p < 0.05, **p < 0.01, n.s. > 0.05 otherwise indicated. Note that wild-type data at 45 °C in **b**–**d**, A455E data at 25, 40, 45, and 50 °C in **c**, and L558S data at 4, 35, 40, 45, and 50 °C in **d** have error bars smaller than the dots. Source data of **a**–**f** are provided as a Source Data file.

We next examined the effect of the A455E and L558S on thermal stability of NBD1-folding intermediates by subjecting ribosome-bound nascent polypeptides to progressive thermal denaturation. In control experiments we showed that with the acceptor dye located at Thr389 (CFP fusion site) (Supplementary Fig. 3a), CFP-NBD1 (full-length NBD1) exhibited a reversible temperature-dependent decrease in CFP fluorescence but no change in FRET (Supplementary Fig. 3b, c). This was expected as the acceptor dye is attached to CFP by a short unstructured tether that is not dependent on NBD1 folding. In contrast, when the acceptor dye was inserted at Arg487 in NBD1 (truncation 654), an irreversible temperature-dependent decrease in FRET was observed (Supplementary Fig. 3d, e), consistent with previous studies showing that NBD1 does not spontaneously refold in vitro^4,5^. We then compared wild-type and mutant ribosome-attached polypeptides truncated at residue 654 and 744. All constructs exhibited a decrease in FRET with increasing temperature, consistent with thermal denaturation from their respective folding states, and both mutants exhibited a lower FRET than wild-type at all temperatures tested (4–50 °C, Fig. 4a–d). At the highest temperatures, differences between wild-type and mutants became less prominent. In addition, the NBD1-folding intermediate (truncation 654), but not full-length NBD1, showed a slightly greater temperature dependence between wild-type and mutant polypeptides with a transition temperature above 35–40 °C (Fig. 4e, f). Overall, temperature-dependent changes in FRET exhibited similar slopes and occurred over the same temperature range for wild-type and mutant polypeptides, suggesting that the mutations change the conformational profile of the folding intermediate without a major change in thermal stability.

### NBD1 transient folding defect is linked to CFTR trafficking

Finally, we applied a genetic approach to test whether restoring the cotranslational folding pathway was possible, and if so, whether it would correct the final folding outcome and restore CFTR trafficking. Here, we took advantage of the numerous suppressor mutations previously shown to improve F508del CFTR trafficking^54,55^. We specifically focused on a proline substitution at residue Ser492 (S492P), which is predicted to increase rigidity between the N-terminal and the α-helical subdomains^56^, and a well-known suppressor mutation I539T^57^, which increases thermal stability of F508del NBD1.

**Fig. 5:**
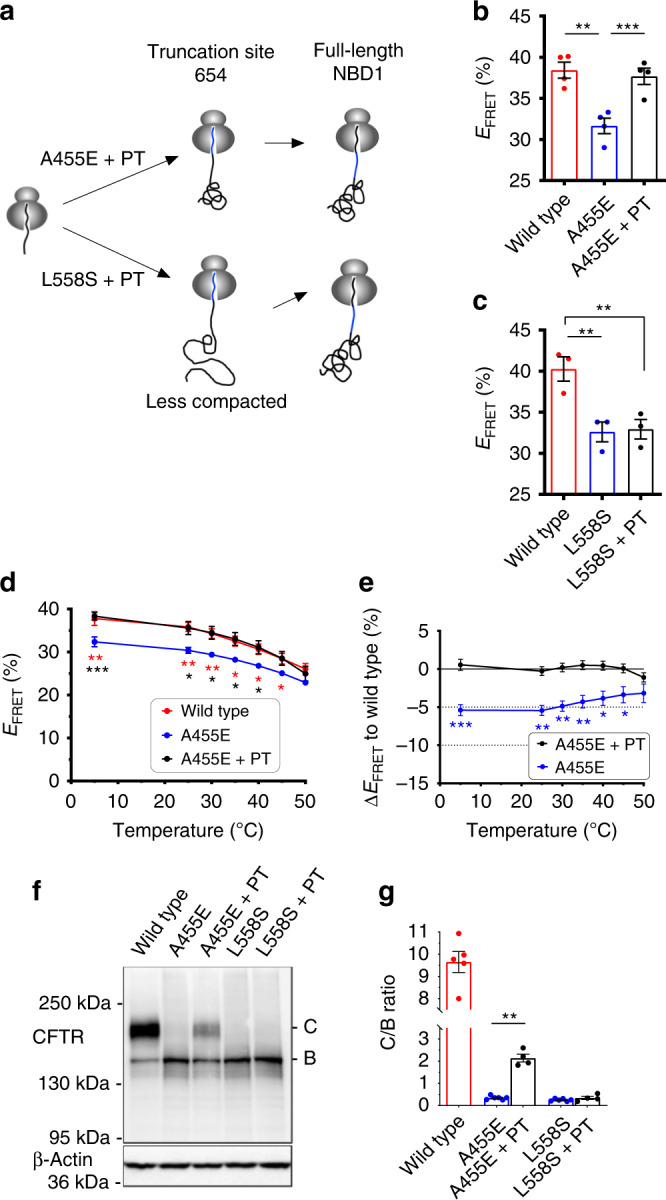
Genetic correction of cotranslational folding also restores CFTR trafficking. **a**–**c** S492P & I539T (PT) suppressor mutations restore FRET efficiency of the folding intermediate (truncation at 654, acceptor dye at Arg487) for A455E **b** but not L558S **c**. Each dot represents data from an independent experiment. Each bar shows mean ± SEM, *n* = 3 or 4 independent experiments. Two-tailed student’s *t* test, asterisk *p* < 0.05, double asterisk *p* < 0.01, triple asterisk *p* < 0.001, n.s. > 0.05 otherwise indicated. **d**, **e** Temperature-dependent FRET efficiencies of wild-type (red line), A455E (blue line), and A455E + PT (black line). Ribosome-bound CFP-NBD1 polypeptides were truncated at residue 654 (acceptor dye at Arg487). The difference between wild-type and mutants in **e** were calculated from the data in **d**. Data are mean ± SEM, *n* = 4 independent experiments. Two-tailed student’s *t* test comparing wild-type and A455E (red asterisks), A455E and A455E + PT (black asterisks), or wild-type and A455E + PT (n.s.) in **d** and one-tailed student’s *t* test in **e**, asterisk *p* < 0.05, double asterisk *p* < 0.01, n.s. > 0.05 otherwise indicated. Note that some points have error bars smaller than the dot. **f**, **g** Immunoblot of CFTR A455E or L558S and A455E or L558S plus PT mutations expressed in human embryonic kidney (HEK) 293 cells showing correction of folding defect for full-length CFTR A455E but not L558S. Uncropped blots in Source Data. Each dot represents data from an independent experiment. Each bar shows mean ± SEM, *n* = 4–6 independent experiments. Two-tailed student’s *t* test, double asterisk *p* < 0.01, n.s. > 0.05 otherwise indicated. Source data of **b**–**g** are provided as a Source Data file.

As shown in Fig. 5a–c, introduction of S492P and I539T (PT) into NBD1 (truncation 654). completely restored FRET for A455E to the level observed for wild-type polypeptides but had no effect on the L558S mutant. In addition, combination of the PT suppressor with A455E restored thermal sensitivity of nascent polypeptides to a level that was indistinguishable from wild type (Fig. 5d, e). We then tested whether correction of the cotranslational folding effect would translate to the folding outcome of full-length CFTR. As shown in Fig. 5f, g, incorporation of the PT suppressor in full-length CFTR partially restored A455E CFTR trafficking in HEK293 cells as indicated by an increase in fully glycosylated band C protein to ~20% of wild-type levels, whereas no improvement was observed in the processing of L558S CFTR. Partial restoration of A455E CFTR trafficking observed here is analogous to the effect of other suppressor mutations that effectively restore F508del NBD1 stability but have a relatively minor effect on CFTR trafficking^8,58,59^, and suggest that the cotranslational defect is not the only folding defect induced by the A455E mutation. Taken together, these results show that genetic complementation by suppressor mutations can overcome defects associated with transient folding intermediates and thereby partially restore the native folding landscape and final folding outcome.

## Discussion

In this study, we show that disease-causing missense mutations in the CFTR chloride channel disrupt specific steps of the cotranslational folding pathway. Specifically, we demonstrate that (i) mutant NBD1-folding intermediates are less compact than their wild-type counterparts; (ii) two mutants, A455E and L558S, exert detectable effects during a brief window of synthesis that involves the coordinated folding of the α-helical subdomain and the β-sheet core; (iii) the effect of ribosome attachment initially accentuates the folding defect by delaying α-helical subdomain folding, and subsequently facilitates domain compaction as synthesis of NBD1 was completed; and (iv) genetic suppressor mutations that restore cotranslational folding also partially restore trafficking of full-length CFTR. These results indicate that disruption of protein folding by disease-causing mutations begins early during synthesis and suggest that transient folding intermediates provide potential targets for restoring folding in human disease.

The de novo protein folding landscape is characterized by folding intermediates, likely comprised of structural ensembles, that exist transiently and are sequentially remodeled as the nascent polypeptide progressively emerges from the ribosome exit tunnel^17,18,24,47,60^. With the caveat that some secondary structure (e.g., alpha-helices) may form within the ribosome exit tunnel^20,61,62^, structure features of the nascent polypeptide are dictated primarily by the peptide region that becomes cytosolically exposed at any given length^17,18,60,63^. Consistent with this, the N-terminal domain of HemK was shown to rapidly transition into a native-like structure after emerging from the ribosome^27^, consistent with results reported for β-barrel fluorescent proteins^60^. Molecular simulations of large single-domain proteins also predict that fast folding kinetics and/or transient translational pauses can improve cotranslational folding by avoiding kinetically stable non-native interactions with C-terminal residues^64^. Similarly, synonymous codon changes can enhance in vivo degradation without substantially changing activity/function and thermal stability^34^. An extreme example reported by Sander et al.^32^ used a tandem fluorescent protein approach wherein alternate stable structures were generated from the same polypeptide sequence based on the order in which cotranslational folding occurred. Therefore, the folding pathway is an important determinant of the final folded state. While it is well known that disease-causing mutations can impact the stability and/or structure of fully synthesized CFTR^6,65^, our results indicate that mutations can also exert their effect by disrupting intermediates along the folding pathway and thereby alter folding outcome.

The FRET-based approach used here provides a unique opportunity to evaluate transient folding intermediates, and several aspects of this system support our interpretation that changes in FRET reflect a conformational change in the structural ensemble of the nascent polypeptide^48,49^. First, all parameters of the experimental system were held constant with the exception of the mutant residue, and each experiment was carried out in parallel with the wild-type sequence to allow a direct comparison between wild-type and mutant nascent polypeptides. Second the absolute CFP fluorescence intensity (pps/nm) remained constant (as reported previously^60^) regardless of NBD1 chain length making it highly unlikely that the ribosome, the nascent NBD1 polypeptide, or other cellular factors affect FRET efficiency by altering CFP folding. Third, multiple sites for acceptor probe incorporation (described elsewhere) were evaluated and chosen so as not to grossly perturb NBD1 folding^17,18^. Overall, this approach enabled us to directly compare measurements made under identical experimental conditions where differences in FRET can be ascribed to the change of a single amino acid in the nascent polypeptide.

An important caveat of this system is that the readout for peptide compaction is limited to changes that can be detected by location of the donor and acceptor probes. The probe sites therefore restrict our ability to determine the precise nature of a structural defect and to identify changes that have minimal effect on the distance between these specific probes. Thus, the apparent resolution of the NBD1 folding defect observed at longer chain lengths as shown in Fig. 2 may be due to: (i) the inability of the probe sites to detect a residual folding abnormality^17,18^, (ii) a decrease in domain stability with minimal structural change, similar to that observed for crystal structures of F508del^8,66^ (albeit containing stabilizing site suppressor mutations), or (iii) subtle structural changes that may disrupt later folding events such as domain–domain assembly, which are known to be critical for CFTR trafficking^2^. It is also possible that in this system, the isolated wild-type NBD1 domain does not reach a completely folded native structure, thereby allowing FRET values to converge at longer chain lengths. Further studies using different approaches, for example NMR studies of nascent translation intermediates and/or high resolutions structures of A455E and L558S NBD1, will be needed to generate a more complete understanding of this process.

We do know, however, that NBD1 subdomains undergo a coordinated, stepwise folding process that is critical for NBD1 and full-length CFTR to achieve a mature conformation^17,18^. For example, the N-terminal subdomain folds rapidly and independently, whereas folding of the α-helical subdomain and β-sheet core is tightly coupled to translation and coordinated by biosynthetic machinery^18^. Previous studies using stalled ribosome-attached nascent chain complexes (RNCs) have shown that the ribosome can exert destabilizing effects on nascent polypeptide relative to the same peptide region free in aqueous solution^22,28–30^. For CFTR, the net result of this destabilizing effect is to delay compaction of the α-helical subdomain until the β-sheet core is synthesized and thereby ensure that subdomains fold efficiently and in the proper temporal order to avoid off-pathway intermediates^18^. These results are in good agreement with recent studies by Alexander et al.^30^, where ribosome-mediated delay of calerythrin N-terminal domain folding also facilitated overall folding efficiency.

Here, we show that the destabilizing effect of the ribosome exacerbates the disruptive effects of A455E and L558S mutations by further delaying α-helical subdomain folding (Fig. 2). Somewhat paradoxically, ribosome attachment also promoted and was required for domain compaction as NBD1 synthesis was completed (Fig. 3c, d). Thus the ribosome influences both normal and abnormal folding intermediates, and for A455E and L558S alters the normal progression (and timing) of NBD1 cotranslational folding events. It is important to consider that the kinetics of NBD1 de novo folding in the cell, relative to its native translation rate, could add an additional layer of complexity if the nascent folding intermediates are not in equilibrium during active peptide elongation^30^. In this context, the ribosome can cooperate with cellular chaperones to decelerate, accelerate and/or reverse domain folding to improve folding outcomes^42^. Reticulocyte lysate is an abundant source of HSP40, HSP70, HSP90, and other physiologic cellular chaperones that alter the rate of de novo folding and likely make an important contribution to our findings^25,37–41,67,68^. For technical reasons, we were not able to determine the effect of cellular chaperones on NBD1 folding in this study, and additional work is therefore needed to determine more precisely how these and other factors might influence the folding events studied here.

An unexpected finding was that NBD1 cotranslational folding defects were observed during a relatively brief window of synthesis, specifically as α-helix H5 to β-strand S7 emerge from the ribosome, and were followed by domain compaction as the remaining NBD1 residues became available (Fig. 2). One possibility, given the very high contact order of NBD1^17,69^, is that mutations of this nature exert their greatest disruptive effect when limited folding information (chain length) is available to stabilize nascent structure. The magnitude of the disruptive effect could therefore be decreased by stabilizing interactions that are provided by C-terminal residues during late stages of NBD1 synthesis^17,18,24,47,60^. Yet despite the apparent compaction of the domain as measured by FRET (Fig. 2c–f), some residual folding defect must still remain in the full-length CFTR protein as demonstrated by its poor processing efficiency (Fig. 2a). Given the multidomain structure of CFTR, the precise nature of this folding defect is likely complex. PT suppressor mutations, which restore the in vitro cotranslational defect at short NBD1 chain lengths, only partially restore folding of the full-length protein in cells. Thus, in a manner analogous to F508del, cotranslational misfolding of A455E likely results in a local perturbation in NBD1 that is undetected by FRET, which impacts an additional subsequent alteration (e.g., domain–domain interactions) in the full-length protein.

CFTR processing is disrupted by multiple mutations that act via different mechanisms. For the most common variant, F508del, second site suppressor mutations have been identified that either stabilize NBD1^55,57,59^ or correct defects in the NBD1–ICL4 interface. In each case, correcting a single defect only partially restores CFTR maturation in the cell^6,8^, and efficient maturation requires correcting both defects simultaneously^7,8^. This is also seen with pharmacological correction where the relatively weak effects of Lumacaftor and Tezacaftor, are greatly augmented by addition of a second corrector acting on a different folding defect. Consistent with these findings, PT suppressor mutations that correct the A455E NBD1 cotranslational folding defect only partially restore A455E CFTR trafficking in cells (Fig. 5), indicating that like F508del, A455E likely induces multiple folding defects. In addition, although A455E and L558S act at a similar stage of synthesis, PT mutations suppressed the cotranslational defect and restored CFTR trafficking only for A455E (Fig. 5), likely because introduction of a negatively charged residue into the NBD1 core (A455E) induces a different structural perturbation than a Leu-to-Ser substitution in the α-helical subdomain (L558S). The finding that both mutations act during the same window of synthesis, supports the previous hypothesis that folding of the α-helical and β-sheet core is finely tuned and particularly sensitive to disruption^18^.

Taken together, our results indicate that cotranslational folding intermediates play an important role in the underlying mechanisms of de novo protein folding, and raise the possibility that they contribute to the molecular pathology of protein folding disorders. Although additional studies are needed to determine whether cotranslational folding intermediates might be druggable targets, genetic suppression of the cotranslational defect and the observed cotranslational effects of Lumacaftor on CFTR folding suggest that small molecules acting on such intermediates could potentially help restore CFTR processing. Thus, our findings may aid in the understanding and development of treatment approaches for small molecule correction in CF and other protein-folding disorders.

## Methods

### Plasmids

pSP64-eCFP-NBD1 containing Amber stop codons (TAG) at CFTR residues Thr389, Arg487, and Asp567, pSP64-non-fluorescent CFP-NBD1 fusion constructs^17,18^, and pcDNA3-CFTR^52^ were used in this study. For cloning, CF-causing mutations (L441P, A455E, S492F, I507del, F508del, V520F, G551D, L558S, A559T, R560K, and R560T), a non-disease-causing mutation M470V, and suppressor mutations (S492P and I539T) were individually introduced into pSP64-eCFP-NBD1-R487TAG^17,18^, pSP64-eCFP-NBD1-D567TAG^17,18^, or pcDNA3-CFTR^52^ by PCR overlap extension using complementary sense and antisense oligonucleotides (primers# 1–28) listed in Supplementary Table 1. The CFTR2 database (https://www.cftr2.org) was referred to design the oligonucleotides. PCR fragment containing the mutation L441P, A455E, M470V, S492F, I507del, F508del, or V520F was subcloned into PstI and SphI sites of pSP64-eCFP-NBD1-R487TAG/D567TAG^17,18^. PCR fragment containing the mutation L558S, A559T, R560K, or R560T was subcloned into SphI and SpeI sites of pSP64-eCFP-NBD1-R487TAG/D567TAG^17,18^. PCR fragment containing the mutations S492P and I539T (PT) was subcloned into PstI and SpeI sites of pSP64-eCFP-NBD1-R487TAG^17,18^. PCR fragment containing the mutations A455E, L558S, A455E + PT, or L558S + PT was subcloned into BsrGI and HpaI sites of pcDNA3-CFTR^52^. All cloned PCR fragments were verified by DNA sequencing. Truncated cDNA for in vitro transcription was generated by PCR amplification using appropriate oligonucleotides (primers# 29–41) listed in Supplementary Table 2.

### In vitro transcription and translation

Noncapped RNA transcripts were synthesized from PCR-amplified DNA templates (15 ng/μl) in a solution containing 80 mM HEPES-NaOH (pH 7.5); 16 mM MgCl_2_; 2 mM spermidine; 3 mM each ATP, CTP, UTP, and GTP; 10 mM DTT; 0.2 U/μl RNase inhibitor; and 5 μg/ml SP6 RNA polymerase at 40 °C for 2 h. RNA was precipitated with 3 M LiCl and 20 mM ethylenediaminetetraacetic acid (EDTA) at −20 °C for 1 h and washed three times with 70% (v/v) ethanol, followed by centrifugation at 16,000 × *g* at 4 °C. The RNA pellet was resuspended in RNase free H_2_O and stored at −80 °C.

Four 250 μl in vitro translation reactions were prepared in parallel in reactions containing 60 ng/μl purified RNA, 40% rabbit reticulocyte lysate (RRL), 20 mM HEPES-KOH (pH 7.6), 100 mM KOAc, 1.6–2.0 mM Mg(OAc)_2_, 50 μM each of 20 amino acids, 1 mM ATP, 1 mM GTP, 15 mM creatine phosphate, 2 mM DTT, 0.15 mM spermidine, 20 ng/μl bovine tRNA, 40 ng/μl creatine kinase, 0.12 U/μl RNase inhibitor, 0.2–2 μM RNA aptamer^70^, and 1 μM synthetic amber suppressor tRNA (either [^14^C]Lys-tRNA^amb^ or εN-7-nitrobenz-2-oxa-1,3-diazol (εNBD)-[^14^C]Lys-tRNA^amb^)^17^. To generate matched samples for fluorescence spectroscopy, CFP donor only (D)—translated in the presence of [^14^C]Lys-tRNA^amb^, Donor + Acceptor (D + A)—translated in the presence of εNBD-[^14^C]Lys-tRNA^amb^, and two “blank” (control) reactions using non-fluorescence eCFP lacking a UAG codon translated in the presence of either [^14^C]Lys-tRNA^amb^ or εNBD-[^14^C]Lys-tRNA^amb^ (BD and BDA) were prepared as described in Supplementary Figure 1b. Blank samples were used to quantify the concentration of RNCs and obtain ^14^C-corrected fluorescence intensity in solution-based FRET measurement. An RNA aptamer that inhibits translation termination factors (eRF1/eRF3) was added (0.2–2 μM) to achieve similar read-through efficiencies of the UAG codon for D and D + A translation reactions^18,70^. Translation reactions were incubated at 24 °C for 8 min prior to addition mRNA, suppressor tRNA, and RNA aptamer, and then at 24 °C for 72 min (to enable CFP folding and maturation^60^).

### Compensation for effects of in vitro translational stalls

If read-through efficiency at the UAG codon differs for D and D + A samples in single experiments, then stalled RNCs in D and D + A samples can introduce affect in FRET. This is because the relative fraction of stalled polypeptides (with zero FRET) in the sample, is dependent on both the number of stalls and the number of polypeptides that actually read-through the stop codon^18^. To eliminate this potential artifact, only samples with D/DA ratio between 0.80 and 1.20, or 0.85 and 1.15 were used for R487TAG or D567TAG constructs, respectively.

### Fluorescence measurements

Intact RNCs were isolated from translation reactions at 4 °C by size exclusion column chromatography (Sepharose CL-6B) equilibrated in buffer containing 40 mM HEPES-KOH (pH 7.6), 100 mM KOAc, and 10 mM MgCl_2_, and the ribosome concentration (A_260_) in each sample was normalized by addition of buffer. CFP fluorescence emission spectra (λex = 430 nm, λem = 450–600 nm, 1 nm intervals) obtained from purified RNCs were measured using a Fluorolog 3-22 fluorometer (HORIBA Jobin Yvon, Edison, NJ) at 25 °C unless otherwise specified. The five highest peak emission intensities (~475 nm) were averaged and used for subsequent calculation. Ribosome release was performed by adding 200 μg/ml RNase A in column buffer plus 3 mM ATP, which effectively releases all nascent chains from the ribosome with 2 min^17^. At the end of measurement, the concentration of polypeptide in D and D + A samples was determined by ^14^C scintillation counting using the following equation:

1 $$\left[ {{\mathrm{RNC}}} \right] = \left( {{\mathrm{cpm}}_{\mathrm{S}} - {\mathrm{cpm}}_{\mathrm{B}}} \right)/\left( {{\mathrm{CE}}\;x\;{\mathrm{SA}}\;x\;{\mathrm{vol}}} \right)$$

where [RNC] is the concentration of RNCs (in nM), cpm_S_ is ^14^C counts/min of D or D + A sample, and cpm_B_ is ^14^C counts/min of blank (BD or BDA), respectively. CE is counting efficiency (estimated at 95% for ^14^C), SA is the specific activity of [^14^C]Lys in dpm/pmol, and vol is the volume of sample in ml.

FRET efficiency was then calculated by the acceptor-dependent decrease in CFP fluorescence intensity, from the following equation:

2 $${\mathrm{E}}_{{\mathrm{FRET}}} = 1 - {\mathrm{F}}_{{\mathrm{DA}}}/{\mathrm{F}}_{\mathrm{D}}$$

where F_DA_ and F_D_ are the net ^14^C-corrected fluorescence intensity per nM nascent chain polypeptide in D + A and D samples, respectively. Note that fluorescence intensity of the acceptor is negligible in our sample (under these conditions)^17,18^.

Each experiment was performed with a matched pair of wild-type and mutant constructs, therefore, controlling for day-to-day variation in FRET efficiency, and allowing a direct comparison of differences in FRET efficiency between wild-type and mutant constructs to be evaluated directly at specific stage of synthesis.

### Immunoblotting

Human embryonic kidney (HEK) 293 cells (ATCC, catalog# CRL-1573) were grown at 37 °C under 5% CO_2_ in Dulbecco’s modified Eagle’s medium (Invitrogen) supplemented with 10% fetal calf serum (Thermo Scientific) and penicillin/streptomycin (Invitrogen). In total, 5 × 10^5^ cells were seeded in six-well plates and transfected 1 day later with equal amounts (2.5 μg) of pcDNA3-CFTR vector. Cells were harvested 48 h after transfection by lysis for 20 min in 600 μl of ice-cold RIPA buffer (20 mM HEPES-NaOH/pH 7.5, 150 mM NaCl, 1 mM EDTA, 1% Triton X-100, 0.1% SDS, 0.5% sodium deoxycholate) containing complete protease inhibitor mixture (Roche Applied Science). Cell lysate was separated by sodium dodecyl sulfate polyacrylamide gel electrophoresis, transferred to polyvinylidene difluoride membrane (Millipore), and immunoblotted using the following primary antibodies: (1) mouse anti-CFTR M3A7 (Millipore, Cat# 05-593, Lot# 2652963, 1:2000 dilution), or (2) rabbit anti-β-actin C4 (Santa Cruz Biotech., Cat# sc-47778, Lot# B1914, 1:2000 dilution) and secondary antibodies: (3) goat anti-mouse IgG (H + L)-HRP conjugate (Bio-Rad, Cat# 1706516, 1:5000 dilution), or (4) goat anti-rabbit IgG-HRP (Santa Cruz Biotech., Cat# sc-2030, 1:5000 dilution). Blots were imaged using the ChemiDoc XRS + System (Bio-Rad) and analyzed using accompanying image analysis software. Uncropped blots are available in the Source Data file.

### Reporting summary

Further information on research design is available in the Nature Research Reporting Summary linked to this article.

## Supplementary information

Supplementary Information

Peer Review File

Reporting Summary

## Data Availability

Data supporting the findings of this manuscript are available from the corresponding author upon reasonable request. A reporting summary for this Article is available as a Supplementary Information file. Source data are provided with this paper.

## Source data

Source Data
