# Supplementary material for: CFTR trafficking mutations disrupt cotranslational protein folding by targeting biosynthetic intermediates
Source: Nat Commun. 2020 Aug 26;11:4258. doi: 10.1038/s41467-020-18101-8 (PMC7450043; doi:10.1038/s41467-020-18101-8)
Supplement: Supplementary file 1 — Supplementary Information [file 41467_2020_18101_MOESM1_ESM.pdf]

Supplementary Information

CFTR trafficking mutations disrupt de novo protein folding by targeting biosynthetic intermediates

Shishido H. et al.

This pdf file includes:

Supplementary Figure 1

Supplementary Figure 2

Supplementary Figure 3

Supplementary Table 1

Supplementary Table 2

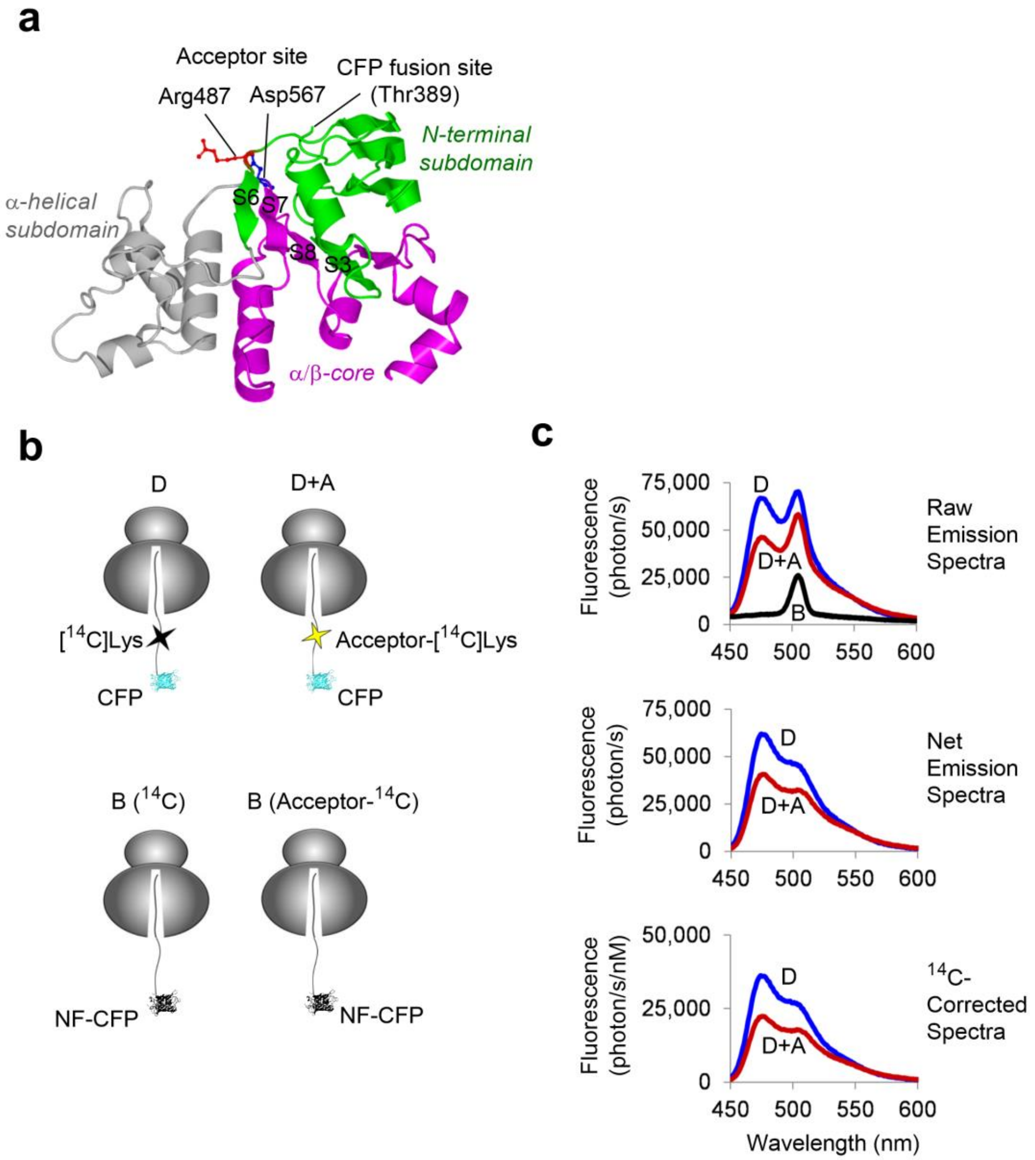

Supplementary Figure 1. Experimental FRET assay setup. (**a**) Location of CFP fusion site (Thr389) and acceptor dyes (Arg487 and Asp567) in the NBD1 crystal structure (PDB: 5UAK). (**b**) Schematic of in vitro generated ribosome-nascent chain complexes (RNCs) showing Donor only (D) and Donor+Acceptor (D+A) translated in the presence of [^{14}C]Lys- tRNA^{amb} or εNBD-[^{14}C]Lys-tRNA^{amb}, respectively. Blank translations [B (^{14}C or Acceptor-^{14}C)] were programmed with transcript encoding non-fluorescent (NF) CFP- lacking a UAG codon but translated in the presence of [^{14}C]Lys-tRNA^{amb} or εNBD-[^{14}C]Lys-tRNA^{amb}, respectively. (**c**) Typical CFP fluorescence emission spectra obtained from CFP-NBD1-R487UAG truncated at residue 654 for D, D+A, and B samples, respectively (λex = 430 nm, λem = 450-600 nm). Blank spectra are superimposed. Emission peak at 510 nm is from water Raman scatter. Net CFP emission spectra obtained after blank subtraction. ^{14}C-corrected emission spectra (photon/second/nM protein) calculated from [^{14}C]Lys content based on ^{14}C scintillation counting using the Equation (1) as described in Methods. FRET efficiency is calculated as reported previously[17,18] based on the decrease in donor fluorescence intensity due to the presence of the acceptor probe using the Equation (2) as described in Methods. Source data of **c** are provided as a Source Data file.

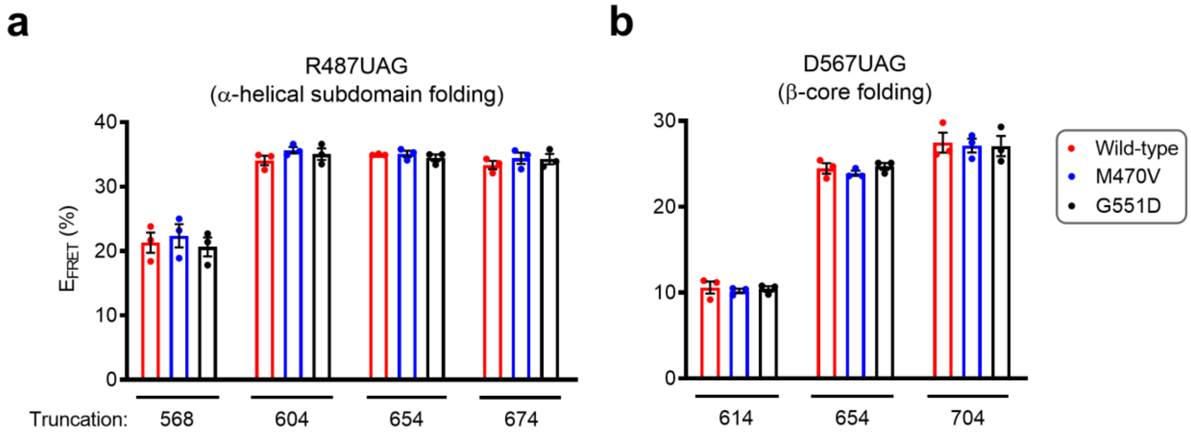

Supplementary Figure 2. A non-disease-causing mutation, M470V, and a non-NBD1-misfolding mutation, G551D, do not disrupt NBD1 cotranslational folding. (**a**,**b**) Length dependent FRET efficiencies for wild type (red), A455E (blue), or L558S (black) obtained from ribosome-attached CFP-NBD1 constructs with acceptor dye located at R487UAG (**a**) or D567UAG (**b**) for each truncation site indicated. Each dot represents data from an independent experiment. Each bar shows mean ± SEM, n=3 independent experiments. Two-tailed student's t-test comparing wild-type and A455E or L558S, n.s. > 0.05 otherwise indicated. Source data of **a** and **b** are provided as a Source Data file.

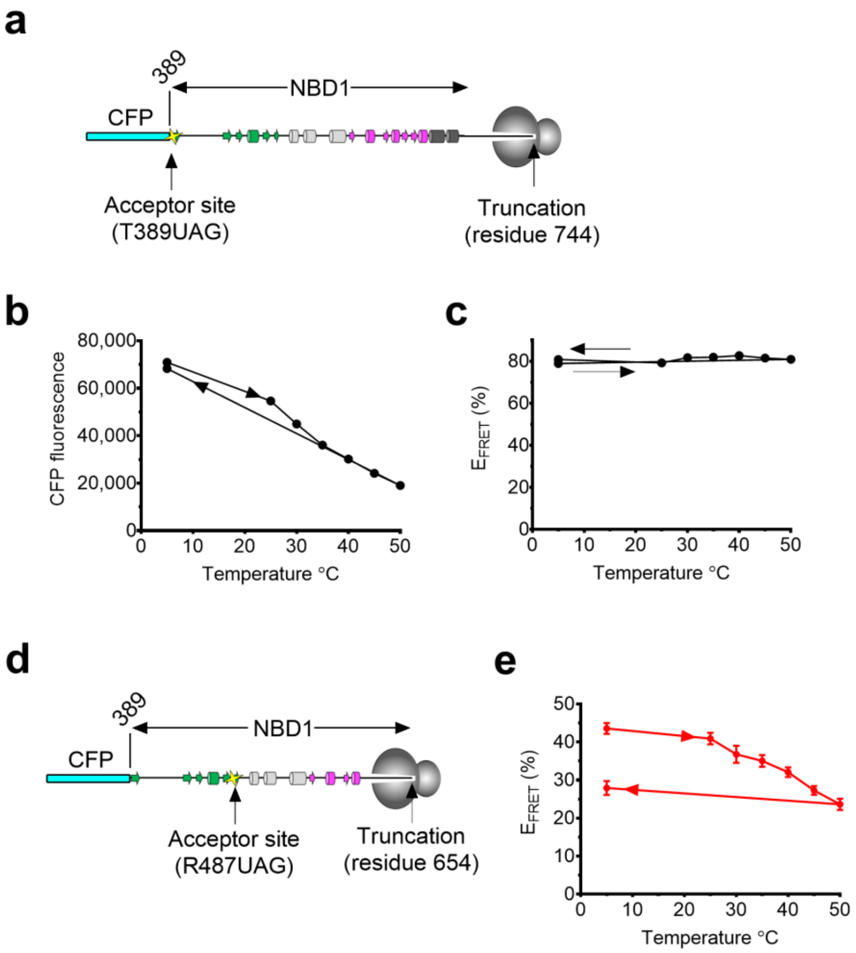

Supplementary Figure 3. Thermal denaturation of ribosome-attached NBD1 folding intermediates. (**a**) Schematics of ribosome-attached CFP-NBD1 showing approximate location of acceptor dye at residue Thr389 and truncation site at residue 744 used in **b** & **c**. (**b**) CFP fluorescence intensity shows fully reversible temperature-dependence. (**c**) FRET efficiency of ribosome-attached CFP-NBD1 with an acceptor dye at Thr389 (CFP fusion site) showing FRET is not dependent on temperature because proximity of probes is independent of NBD1 folding. (**d**,**e**) FRET efficiency of ribosome-attached CFP-NBD1 truncated at residue 654 with an acceptor dye at Arg487 (wild-type) shows temperature-dependent decrease in FRET (consistent with nascent polypeptide denaturation that is not reversible upon cooling). Data are mean ± SEM (n=3 independent experiments). Source data of **b**, **c**, and **e** are provided as a Source Data file.

Supplementary Table 1. Primers used for cloning

Primer #	Mutation	Forward/reverse	Sequence
1	L441P	Forward	ACTCCTGTCCCGAAAGATATTAATTTCAAGATAG
2	L441P	Reverse	ATTAATATCTTTCGGGACAGGAGTACCAAGAAG
3	A455E	Forward	TGTTGGAGGTTGCTGGATCCACTGG
4	A455E	Reverse	CAGTGGATCCAGCAACCTCCAACAACTGTCCTCTTTC
5	M470V	Forward	TCACTTCTAATGGTGATTATGGGAGAACTGG
6	M470V	Reverse	TCCCATAATCACCATTAGAAGTGAAGTCTTGC
7	S492F	Forward	TCATTCTGTTTCCAGTTTTCCTGGATTATGC
8	S492F	Reverse	CCAGGAAAACTGGAAACAGAATGAAAT
9	S492P	Forward	ATTTCATTCTGTCCTCAGTTTTCCTG
10	S492P	Reverse	GGAAAACTGAGGACAGAATGAAAT
11	I507del	Forward	AAAGAAAATATCTTTGGTGTTTCCTATGATG
12	I507del	Reverse	GGAAACACCAAAGATATTTTCTTTAATGGTGCC
13	F508del	Forward	GAAAATATCATTGGTGTTTCCTATGATGAATATAG
14	F508del	Reverse	ATAGGAAACACCAATGATATTTTCTTTAATGGTGCC
15	V520F	Forward	TACAGAAGCTTCATCAAAGCATGCCAACTAG
16	V520F	Reverse	GCATGCTTTGATGAAGCTTCTGTATCTATATTC
17	I539T	Forward	GAGAAAGACAATACAGTTCTTGGAGAAGGTGG
18	I539T	Reverse	TCCAAGAACTGTATTGTCTTTCTCTGCAAAC
19	G551D	Forward	CACTGAGTGGAGATCAACGAGCAAGAATTTCTTTAGC
20	G551D	Reverse	CTTGCTCGTTGATCTCCACTCAGTGTGATTCC
21	L558S	Forward	AGAATTTCTTCAGCAAGAGCAGTATACAAAG
22	L558S	Reverse	TACTGCTCTTGCTGAAGAAATTCTTGCTCGTTG
23	A559T	Forward	ATTTCTTTAACAAGAGCAGTATACAAAG
24	A559T	Reverse	GTATACTGCTCTTGTTAAAGAAATTCTTGCTCG
25	R560K	Forward	TCTTTAGCAAAAGCAGTATACAAAGATGC
26	R560K	Reverse	TTTGTATACTGCTTTTGCTAAAGAAATTCTTGC
27	R560T	Forward	TCTTTAGCAACAGCAGTATACAAAGATGC
28	R560T	Reverse	TTTGTATACTGCTGTTGCTAAAGAAATTCTTGC

Supplementary Table 2. Primers used to prepare PCR-amplified DNA templates for in vitro transcription

Primer #	Truncation site	Forward/reverse	Sequence
29	For all sites	Forward	AGAGGATCTGGCTAGCGATG
30	550	Reverse	GTCACACTCAGTGTGATTCCACC
31	568	Reverse	GTCACATCAGCATCTTTGTATAC
32	584	Reverse	GTCACTTCTGTTAAAACATCTAGGTATC
33	604	Reverse	GTCACGACCAAAATCCTAGTTTTGTTAG
34	614	Reverse	GTCACAGCTTTCTTTAAATGTTC
35	624	Reverse	GTCACGCTACCTTCATGCAAAAT
36	634	Reverse	GTCACGAGTTCTGAAAATGTCCC
37	654	Reverse	GTCACAAATTGGTCGAAAGAATC
38	664	Reverse	GTCACAGTTAGGATTGAATTTCTTCTTTC
39	674	Reverse	GTCACTCCTTCTAATGAGAAACG
40	704	Reverse	GTCACATTGAGAATAGAATTCTTCCTTTTTTC
41	744	Reverse	GTCACCTCAGAATCTGGTACTAAGG
