# Supplementary material for: CFTR trafficking mutations disrupt cotranslational protein folding by targeting biosynthetic intermediates
Source: Nat Commun. 2020 Aug 26;11:4258. doi: 10.1038/s41467-020-18101-8 (PMC7450043; doi:10.1038/s41467-020-18101-8)
Supplement: Supplementary file 3 — Reporting Summary [file 41467_2020_18101_MOESM3_ESM.pdf]

Corresponding author(s): William R. Skach
Last updated by author(s): Jul 22, 2020

Reporting Summary

Nature Research wishes to improve the reproducibility of the work that we publish. This form provides structure for consistency and transparency in reporting. For further information on Nature Research policies, see our Editorial Policies and the Editorial Policy Checklist.

Statistics

For all statistical analyses, confirm that the following items are present in the figure legend, table legend, main text, or Methods section.

n/a	Confirmed	
☐	☒	The exact sample size (*n*) for each experimental group/condition, given as a discrete number and unit of measurement
☐	☒	A statement on whether measurements were taken from distinct samples or whether the same sample was measured repeatedly
☐	☒	The statistical test(s) used AND whether they are one- or two-sided<br>*Only common tests should be described solely by name; describe more complex techniques in the Methods section.*
☒	☐	A description of all covariates tested
☒	☐	A description of any assumptions or corrections, such as tests of normality and adjustment for multiple comparisons
☐	☒	A full description of the statistical parameters including central tendency (e.g. means) or other basic estimates (e.g. regression coefficient) AND variation (e.g. standard deviation) or associated estimates of uncertainty (e.g. confidence intervals)
☐	☒	For null hypothesis testing, the test statistic (e.g. F, t, r) with confidence intervals, effect sizes, degrees of freedom and P value noted<br>*Give P values as exact values whenever suitable.*
☒	☐	For Bayesian analysis, information on the choice of priors and Markov chain Monte Carlo settings
☒	☐	For hierarchical and complex designs, identification of the appropriate level for tests and full reporting of outcomes
☒	☐	Estimates of effect sizes (e.g. Cohen's d, Pearson's r), indicating how they were calculated

Our web collection on statistics for biologists contains articles on many of the points above.

Software and code

Policy information about availability of computer code

Data collection	N/A
Data analysis	GraphPad Prism and Microsoft Excel were used for data analysis. Bio-Rad Image Lab was used for image analysis. MolFeat was used for visualization of protein structures.

For manuscripts utilizing custom algorithms or software that are central to the research but not yet described in published literature, software must be made available to editors and reviewers. We strongly encourage code deposition in a community repository (e.g. GitHub). See the Nature Research guidelines for submitting code & software for further information.

Data

Policy information about availability of data

All manuscripts must include a data availability statement. This statement should provide the following information, where applicable:

- Accession codes, unique identifiers, or web links for publicly available datasets
- A list of figures that have associated raw data
- A description of any restrictions on data availability

The data generated and analyzed in this study are available from the corresponding author upon reasonable request.

Field-specific reporting

Please select the one below that is the best fit for your research. If you are not sure, read the appropriate sections before making your selection.

☒ Life sciences ☐ Behavioural & social sciences ☐ Ecological, evolutionary & environmental sciences

For a reference copy of the document with all sections, see nature.com/documents/nr-reporting-summary-flat.pdf

Life sciences study design

All studies must disclose on these points even when the disclosure is negative.

Sample size

The number of replicates for each experiment is provided. In almost all cases is between three to six as biological replicates to ensure reproducibility of the presented results.

Data exclusions

If translation read-through efficiency at the UAG codon differs for [14C]Lys-tRNA (D) and εNBD-[14C]Lys-tRNA (DA) samples in single experiments, then stalled ribosome-nascent chain complex (RNC) in D and DA samples affect FRET as described in Kim S.J. et al, Science 2015, pp.444-448. Therefore, only samples with D/DA ratio between 0.80 to 1.20, or 0.85 to 1.15 were used for R487TAG, or D567TAG constructs, respectively.

Replication

If translations were inefficient due to excess stalled proteins prior to UAG codon (determined by >100% of UAG-codon-read-through-RNCs) or low yield of RNCs (<0.5nM) caused by low-quality of suppressor tRNA, RNA aptamer, or rabbit reticulocyte lysate (RRL), then measurement of CFP fluorescence is not accurate. This necessitated testing and standardizing the mentioned reagents for experiments reported.

Randomization

All experiments described in this study are biochemical, biophysical, and cell biology experiments in which all samples are defined composition, except for RRL. Variation of RRL due to animal preparation was minimized by testing multiple batches and using only high yield of RRL. Randomization was therefore not performed.

Blinding

The experiments are not blinded as a single researcher mainly designs and executes the experiment, and it was not practical to do for the experiments.

Reporting for specific materials, systems and methods

We require information from authors about some types of materials, experimental systems and methods used in many studies. Here, indicate whether each material, system or method listed is relevant to your study. If you are not sure if a list item applies to your research, read the appropriate section before selecting a response.

Materials & experimental systems

n/a	Involved in the study
☐	☒ Antibodies
☐	☒ Eukaryotic cell lines
☒	☐ Palaeontology and archaeology
☒	☐ Animals and other organisms
☒	☐ Human research participants
☒	☐ Clinical data
☒	☐ Dual use research of concern

Methods

n/a	Involved in the study
☒	☐ ChIP-seq
☒	☐ Flow cytometry
☒	☐ MRI-based neuroimaging

Antibodies

Antibodies used

Anti-CFTR (clone M3A7, Millipore, Cat# 05-583, Lot# 2652963), Anti-beta actin (clone C4, Sata Cruz Biotech., Cat# sc-47778, Lot# B1914), goat anti-mouse IgG (H+L)-HRP conjugate (Bio-Rad, Cat# 1706516), and goat anti-rabbit IgG-HRP (Satna Cruz Biotech., Cat# sc-2030).

Validation

All antibodies were validated by the company. For anti-CFTR, we used a negative control to confirm the absence of signal when no antigen was present.

Eukaryotic cell lines

Policy information about cell lines

Cell line source(s)

HEK293 cell line was originally purchased from ATCC.

Authentication

HEK293 Cells came directly from ATCC in 2013, so previously authenticated.

Mycoplasma contamination | HEK293 Cell line tested negative for mycoplasma contamination.

Commonly misidentified lines (See ICLAC register) | HEK-293 Cells were used to monitor CFTR processing.
