# Supplementary material for: CFTR trafficking mutations disrupt cotranslational protein folding by targeting biosynthetic intermediates
Source: Nat Commun. 2020 Aug 26;11:4258. doi: 10.1038/s41467-020-18101-8 (PMC7450043; doi:10.1038/s41467-020-18101-8)
Supplement: Supplementary file 2 — Peer Review File [file 41467_2020_18101_MOESM2_ESM.docx]

Peer Review Information

**Journal:** Nature Communications

**Manuscript title:** CFTR trafficking mutations disrupt cotranslational protein folding by targeting biosynthetic intermediates

**Corresponding author name(s):** Dr William Skatch

**Editorial notes:**

**Transferred manuscripts**

This manuscript has been previously reviewed at another journal that is not operating a transparent peer review scheme. This document only contains reviewer comments and rebuttal letters for versions considered at *Nature Communications*.

**Reviewer comments & decisions:**

**Reviewer comments, first version:**

Reviewer #1 (Remarks to the Author):

The manuscript by Shishido et al. is a timely and valuable addition to our growing understanding of the impact of co-translational folding on protein production in vivo. In recent years, several new studies have conclusively shown that co-translational folding can alter the mechanism of protein folding in vivo, but to my knowledge Skach’s work on CFTR stands apart in that it directly connects the effects of disease-causing mutations to changes in the conformations of co-translational folding intermediates. This is important, basic research that lays the foundation for (ultimately) designing small molecules and other therapeutic approaches to rectify these folding defects. With only a few exceptions (see below), the experiments are carefully constructed and controlled. I greatly appreciated the authors’ concise yet clear summary of their FRET-based approach and previous results using this method. I have only a few minor suggestions.

1. The manuscript overlooks a really lovely early contribution to the selective effects of mutations (and second-site suppressors) on folding intermediates versus the native structure, from Jon King (Mitraki et al. (1991) Global suppression of protein folding defects and inclusion body formation. Science 253:54). For this reason the use of “novel” in the last sentence of the manuscript Introduction really grates on me. A related note: the page 3 introduction of translation elongation rate effects on co-translational folding should cite Patricia Clark’s 2014 study (ref #53), as it is one of the most clear-cut studies of this phenomenon.

2. Figure 2c-f: Some of the results shown are the average of only two replicates. This appears to apply to the only significantly different FRET result for the effects of A455E on the compaction of the beta-core. Although, this is not entirely clear, as the figure legend also claims that some error bars (for experiments with 3+ replicates) are smaller than the data point size. There should be at least three replicates to support all crucial results. In a situation where three replicates is not possible, the range for the two individual data points collected should be indicated.

3. Figure 5a: The authors’ results do not show that the mutations “affect the thermal stability” of the co-translational folding intermediate. The temperature-dependent FRET changes occur over precisely the same temperature range for the wild-type and mutant constructs, indicating an indistinguishable sensitivity to temperature. The text describing these experiments (pages 12-13) must be revised to correct this. More likely, the mutations change the conformation of the folding intermediate, without changing its stability – for example, by substituting one set of interactions for another.

4. I spent most of the Results section concerned about the in vivo relevance of the changes to these folding intermediates, particularly given the many differences between the cell and the authors’ stalled intermediates, which have been perturbed by the addition of fluorophores and were synthesized in a cell-free translation system (i.e., very slowly, absent crowding, chaperones, etc.). However, I was greatly reassured by the PT suppressor results, particularly that the effects of the PT suppressor mutations observed on ribosomes in vitro corresponded so closely to the effects observed in vivo. For this reason, would the story be more convincing if results from the PT suppression experiments were described earlier? Just an idea.

5. While I very much appreciate the authors’ point that these FRET experiments are technically challenging, the conclusions would be significantly strengthened by showing that other known human mutations (SNPs) in these structural domains that are not disease associated do not lead to the delay in nascent chain compaction. This would significantly strengthen the authors’ claim that the compaction delay is a signature of CFTR misfolding.

6. The Discussion section desperately needs to be re-written. As is, it does a disservice to the significance of the manuscript, as it is too long and mostly repetitive with statements made in the Introduction and Results. Instead, please distill the results and place them in the broader context of our understanding of protein misfolding in general and CFTR in particular. The discussion should elaborate upon and use the manuscript’s results to speculate on major unresolved issues, including (but not limited to) the following:

- The authors show (by both FRET and thermal sensitivity) that the collapse defect caused by the A455E or L558S mutation is resolved for nascent chain lengths >744 aa (Figures 2c-f, 4a, 5b). But in vivo, clearly everything does not “get better”, as these mutations lead to less mature CFTR formed. Why, precisely, do these mutant CFTR proteins fail to mature?

- Why did some of the 10 disease-causing N-terminal subdomain and helical domain mutations not cause a defect in chain compaction? Is this merely due to the FRET pair used here being insensitive to the effects of these mutations (i.e., there IS an effect on collapse, but not in the dimension measured by this FRET pair)? Or are these mutations more likely altering something else about CFTR folding?

- Why do the PT suppressor mutations improve folding only of A455E and not L558S? Particularly if, as the MD results suggest, the two are essentially in contact with one another in the folding intermediate?


Reviewer #2 (Remarks to the Author):

The manuscript by Shishido et al. describes the effect of several CF-causing mutations on the folding process of NBD1. The study used a FRET-based systems previously described and investigated by the authors. By using combination of various truncation, reporter placement and ribosome detachment, the authors identify mutations that modulate the folding process reporters and suggest that it could provide insight on the pathogenesis by several (mainly two) mutants.

The question is highly relevant and the experimental system is elegant. The partial recovery of both FRET signal and protein maturation when introducing reverting mutation is in support of the claim.

However, it is it very difficult to assess whether the findings are indeed relevant to the disease mechanism or mere fluctuation due to the in vitro system.

In particular, one would need some sort of negative control to evaluate whether the (moderate) changes in FRET signal observed in the CF-causing mutants are not seen in the case of non-disease causing mutation. The problem of course is that several disease-causing mutants (that prevent proper CFTR folding) do not affect the FRET pattern.

Of course the most striking one is the absence of change for the F508del, which is known to affect folding and stability of NBD1 and can be reverted by changes in the helical domain of NBD1. The authors have already briefly discussed this in previous publication (Kushoo 2011), speculating that F508del may affect later stages of the folding process. Considering the dramatic effects previously observed for F508del on both NBD1 and full length expression, folding and maturation in various expression systems, this remains very difficult to understand, especially since the FRET based system used here is designed to detect quite detailed changes in the folding process.

The main indication that the changes in signal observed for A455E and L558S are revealing the mechanism of pathogenesis comes from the fact that second site suppressor revert the folding defect and at the same time improves expression for A455E. However, while the FRET signal is identical to wt (suggesting no folding defect), the maturation recovery is limited to 20% (note that the broken axis in Fig6 is misleading). Obviously something else is going on, which is not related to the changes in FRET signal initially detected. A simple interpretation is that the changes of FRET signal are not correlated to the expression defect caused by the mutants. In addition, the same suppressor mutations act efficiently on F508del, although the authors suggest that the folding perturbations are very different.
So it seems very difficult to reconcile the two.

In the end one wonders why a transient folding defect is relevant for the disease considering that extending the truncation point leads to wt-like the FRET folding pattern. Indeed, in the patient’s cell the entire polypeptide is synthesized so it is not obvious why, as stated by the authors, the folding intermediates would play such an important role in the pathogenesis, or at least the ones observed here.

Specific points:
-There is no clear discussion on the possible correlation (or absence of) in the type of mutation studied and the FRET/folding signal. Once again, as F508del is expected to the typical folding deficient mutant and nothing is observed.

-Mutation A455E is a class V mutant, thus where CFTR is functional but produced in insufficient quantities, resulting a mild phenotype. It can be rescued by correctors C3 and C4. The authors should at least have tried those in their assay.

-In Fig1, all data show the FRET signal at truncation 654. According to Fig 2 that is where the strongest difference are seen between wt and either A455E or L558S. Thus a similar figure at different truncations may reveal a different profile. Why was this position chosen for the screening?

-The data from in Fig3 is very difficult puzzling and not well discussed. If folding is properly achieved once reaching position 568, why extending it to 604 would perturb the folded state/intermediate? That seems to go against the idea of co-translational folding. Stating that this is due to the ribosome being around seems like a simplistic statement. Have the authors demonstrated that the ribosome does not interact with the CFP (which could affect FRET signal).

-Fig5: The determination of the thermal stability of NBD1 biosynthetic intermediates of A455E and L558S by this assay is not clear since only the absolute FRET efficiency is different (only for the intermediate state?) but the slopes for both mutants are the same as the wt-NBD1. One would expect a shift at which Efret diminution starts. This further calls for a positive control, mutant with known effect on thermal stability.

Minor points:
- p28: PDB:5UAK is not a crystal structure of NBD1.
- There are a numerous typographical errors, for example
p.11:”truncat*ons”., “determine*4*d
P. 17 : Lumacaftor misspelled 3 times.
p.33: Figure 2a b-acti*c*n
-Fig 3 and fig4 should be grouped obviously.


Reviewer #3 (Remarks to the Author):


The work of Shishido and colleagues uses a cell free translation system and FRET measurements to get insight into the in vivo folding process of the NBD1 domain of the CFTR protein, by analysing gross structural properties of NDB1 fragments of increasing chain length that are ribosome bound. Likewise the system simulates the in vivo co-translantional folding of this particular model protein. The methodologies applied are sound but they are not novel. However, the problem of understanding protein folding in the cell is of crucial importance. Furthermore, the model system investigated has a strong biomedical interest because CFTR is the causing agent of cystic fibrosis. These two points, together with the fact that the article is written for a broad general audience makes the work potentially interesting and appropriate for nature communications. Unfortunately, I cannot recommend its publication in its present form. The reasons are as follows.
1. The focus of the article is on a specific structural intermediate and how its perturbation by mutation disrupts the in vivo folding pathway. To be very honest I don’t think the authors present enough data to back-up their conclusions, at least as they suggest in the abstract and introduction of the paper. Let me explain why. In the protein folding literature a folding intermediate is a conformation populated along the folding pathway. Furthermore, a specific intermediate means that it is possible to structurally characterize it (including via NMR), and clearly specify its structural features (even with atomic detail). However, there is not in this work any piece of data backing up the idea of a specific folding intermediate. In fact the only property accessed is degree of compaction, and the authors highlight as relevant the coupling between the alpha-helical subdomain and the beta-core. Indeed, while reading the paper I noticed many instances mentioning an intermediate but the concept the is not consistent or clear. Let me give some examples:
Abstract – “…a specific structural intermediate…”
Introduction (p5) – “two mutations (A455E and L558S) in different region of NBD1 disrupt compaction of a specific folding intermediate; …”
Introduction (p7) – “The ability to monitor NBD1 folding while the polypeptide remains attached to the ribosome is appealing because it allows access to folding intermediates that exist only transiently during CFTR synthesis. By translating a series of RNA transcripts truncated at defined lengths, it is therefore possible to capture kinetically stalled, transient translation intermediates that reflect snap shots of the folding process, and thus reconstruct the relative compaction of different subdomains as a function of nascent polypeptide length.”
Introduction (p7) – “Hence, a nascent polypeptide truncated at residue 654 examines the effects of mutations on folding intermediates as the β- sheet core is still in the process of acquiring its native structure. This is a particularly critical intermediate state given that α-helical subdomain folding is temporally coupled to β-sheet core formation and its premature folding disrupts NBD1 final structure”
Introduction (p7) – “also decreased compaction of the β-sheet core in this folding intermediate (Fig. 1e). These results indicate that disease-causing mutations can cotranslationally perturb structural intermediates of ribosome-attached nascent polypeptides.”
Results (p12) –“ We next examined the effect of the A455E and L558S on thermal stability of NBD1 intermediates by subjecting ribosome-bound nascent polypeptides to progressive thermal denaturation…”
Discussion (p14) – “transiently disrupt a specific intermediate as detected by FRET during a brief window of synthesis;”
Discussion (p14) – “The de novo protein folding landscape is characterized by sequential folding intermediates (or structural ensembles) that exist transiently and are constantly remodeled as the nascent polypeptide progressively emerges from the ribosome exit tunnel during synthesis”
Discussion (p14) – “The current study shows that disease-related mutations disrupt physical compaction of a specific structural intermediate, thereby directing the nascent polypeptide into off-pathway events that lead to a final misfolded state”

This collection of sentences shows that the authors mention specific structural intermediates, specific folding intermediates, kinetically stalled intermediates, sequential folding intermediates (or structural ensembles (?) – how can this be specific?(!)), as being the same thing. And they clearly are not. So, the first thing the authors should do is to clearly define what an intermediate is in their study. Based on the presented data and to my understanding one can only think of kinetically stalled intermediates (which do not have a specific structure and therefore represent a structural ensemble). If this is the case the conclusions of the paper are much weaker than anticipated by the authors. If this is not the case and the intermediate is specific, then the authors should present data that shows its specific structural traits. Degree of compaction is clearly not enough. I also do not understand how is it that one can conclude from the presented data that the population of the referred ‘intermediate’ is linked to a productive CFTR trafficking.

2. Part of the presented data is based on molecular simulations. As stated in the article the simulations should provide insight into the nature of the transient folding defect (presumably this is the perturbation of degree of compaction). Could the authors specify the intramolecular potential used in their model? Also, could the authors clearly explain the reason why they explore the folding dynamics of NBD1 fragment truncated between residues 389-354? I don’t see why this fragment should provide insight into the experimental data, especially because the experimental data is based on several truncated fragments that are tethered to the ribosome, and the simulations are carried out in bulk conditions. Since tethering clearly constraints the conformational space I don’t see how one can compare the two scenarios (simulations and in vitro), or use the simulations to get insight into structural details taking place during the in vitro folding. It would be much adequate if the simulations could be carried out for one or more fragments of the WT protein tethered to the ribosome, and then for the same fragments with the two selected mutations (i.e. A455E and L558S). A direct comparison of the two model systems could provide more insight on the hypothesis placed by the authors that “these mutations may destabilize a partially collapsed hydrophobic interface that is transiently present during NBD1 translation.” As it stands I don’t see why the simulations are useful. They provide a prediction but the prediction was not tested. On the other hand, since the tethering scenario was not considered, the experimental data can not be interpreted based on the simulations.

3. Other points:

What exactly is the rationale for focusing the study on the A455E and L558S mutations? This is not clear.

There is a recent study (DOI: 10.1039/c6mb00193a) where it is predicted via molecular simulations that substation of residue 492 would restore the folding of F508del-NBD1 and, concomitantly, the surface expression and/or function of F508del-CFTR. This study should be mentioned.

**Author rebuttal, first version:**

Reviewer #1

***Reviewer comment:***

*The manuscript by Shishido et al. is a timely and valuable addition to our growing understanding of the impact of co-translational folding on protein production in vivo. In recent years, several new studies have conclusively shown that co-translational folding can alter the mechanism of protein folding in vivo, but to my knowledge Skach’s work on CFTR stands apart in that it directly connects the effects of disease-causing mutations to changes in the conformations of co-translational folding intermediates. This is important, basic research that lays the foundation for (ultimately) designing small molecules and other therapeutic approaches to rectify these folding defects. With only a few exceptions (see below), the experiments are carefully constructed and controlled. I greatly appreciated the authors’ concise yet clear summary of their FRET-based approach and previous results using this method. I have only a few minor suggestions.*

1. *The manuscript overlooks a really lovely early contribution to the selective effects of mutations (and second-site suppressors) on folding intermediates versus the native structure, from Jon King (Mitraki et al. (1991) Global suppression of protein folding defects and inclusion body formation. Science 253:54). For this reason the use of “novel” in the last sentence of the manuscript Introduction really grates on me. A related note: the page 3 introduction of translation elongation rate effects on co-translational folding should cite Patricia Clark’s 2014 study (ref #53), as it is one of the most clear-cut studies of this phenomenon.*

**Reply:** We thank the reviewer for this suggestion and now acknowledge the important findings of Mitraki et al., cited on page 3 (Ref #13, Science 1991). We had previously overlooked this work since it primarily focused on *suppressor mutations* that inhibited *post-translational* aggregation of the P22 tailspike protein folding intermediates, but agree completely with the relevance of these findings to our studies. Accordingly, we have removed the term “novel” from the sentence on page 5. We also agree that the Clark study describes a seminal finding and now cite Sander I.M. et al., (Ref #34, J Am Chem Soc 2014) in the introduction of translation elongation rate effects on cotranslational folding (page 4).

***Reviewer comment:***

2. *Figure 2c-f: Some of the results shown are the average of only two replicates. This appears to apply to the only significantly different FRET result for the effects of A455E on the compaction of the beta-core. Although, this is not entirely clear, as the figure legend also claims that some error bars (for experiments with 3+ replicates) are smaller than the data point size. There should be at least three replicates to support all crucial results. In a situation where three replicates is not possible, the range for the two individual data points collected should be indicated.*

**Reply:** We agree and have performed additional experiments to show ≥3 independent experimental replicates for all data shown. Changes in the figures are listed below.

- A559T in Fig. 1e;
- Truncations residues 500 and 550 in Fig. 2c & d;
- Truncations residues 584 in Fig. 2d;
- Truncations residues 634 and 704 in Fig. 2e & f.

2

**Shishido H. et al.	Point by point reply to the reviewer’s comments**

We also describe this point regarding the error bars in the legends of Fig. 2c & d and Fig. 4a & b.

***Reviewer comment:***

3. *Figure 5a. The authors’ results do not show that the mutations “affect the thermal stability” of the co-translational folding intermediate. The temperature-dependent FRET changes occur over precisely the same temperature range for the wild-type and mutant constructs, indicating an indistinguishable sensitivity to temperature. The text describing these experiments (pages 12-13) must be revised to correct this. More likely, the mutations change the conformation of the folding intermediate, without changing its stability – for example, by substituting one set of interactions for another.*

**Reply:** We agree and thank the reviewer for this suggestion. The text has been amended to more accurately describe the results of our thermal denaturation experiments (Fig. 4) in the revised manuscript (pages 12-13).

***Reviewer comment:***

4. *I spent most of the Results section concerned about the in vivo relevance of the changes to these folding intermediates, particularly given the many differences between the cell and the authors’ stalled intermediates, which have been perturbed by the addition of fluorophores and were synthesized in a cell-free translation system (i.e., very slowly, absent crowding, chaperones, etc.). However, I was greatly reassured by the PT suppressor results, particularly that the effects of the PT suppressor mutations observed on ribosomes in vitro corresponded so closely to the effects observed in vivo. For this reason, would the story be more convincing if results from the PT suppression experiments were described earlier? Just an idea.*

**Reply:** We appreciate the reviewer’s concern about the in vitro expression system, and the suggestion to describe the PT suppression experiments earlier in the manuscript., There is no perfect system to investigate the process of de novo protein folding, and every system has its limitations. RRL does provide analysis of synchronized folding intermediates synthesized in the context of native biosynthetic (cellular chaperones^1^) and degradation machinery (ubiquitin proteasome pathway^2^), as well as a limited degree of molecular crowding (~20% cytosol ~80 mg/ml protein) This is now discussed on page 7. We have also shown (Khushoo A. et al., Mol. Cell 2011) that NBD1 generated in vitro folds sufficiently to bind its natural ATP ligand^3^. While we carefully considered the suggestion to move results of the PT suppression experiments earlier, on balance, we felt that the current flow better allows comparison of A455E and L558S (and other) mutations and therefore we prefer to consolidate the in vivo data in Fig.5.

***Reviewer comment:***

5. *While I very much appreciate the authors’ point that these FRET experiments are technically challenging, the conclusions would be significantly strengthened by showing that other known human mutations (SNPs) in these structural domains that are not disease associated do not lead to the delay in nascent chain compaction. This would significantly strengthen the authors’ claim that the compaction delay is a signature of CFTR misfolding.*

**3**

Shishido H. et al.	Point by point reply to the reviewer’s comments

**Reply:** This suggestion was also raised by Reviewer 2. To demonstrate that the observed delay in compaction is a feature specific for pathogenic mutations, we examined two additional well-described CFTR variants, M470V, which alone is not associated with disease, and G551D which causes CF by disrupting CFTR channel gating, but has no effect on folding or trafficking. These variants were chosen in part because the mutant residues, M470 and G551, are relatively close in primary sequence and domain location, to A455E and L558S, respectively. Analysis of a series of truncations for each control variant are now provided in Supplemental Figure 2 and described on page 10. Notably, there was no detectable difference in the observed FRET values from the wild-type, supporting our claim that the observed compaction delay is, indeed, related to certain CFTR misfolding mutants.

***Reviewer comment:***

*6. The Discussion section desperately needs to be re-written. As is, it does a disservice to the significance of the manuscript, as it is too long and mostly repetitive with statements made in the Introduction and Results. Instead, please distill the results and place them in the broader context of our understanding of protein misfolding in general and CFTR in particular. The discussion should elaborate upon and use the manuscript’s results to speculate on major unresolved issues, including (but not limited to) the following:*

**Reply:** In the original version we were deliberately conservative in our interpretations to avoid being criticized for over-speculation, but we fully agree that the study raises numerous implications relating to effects of disease-causing mutations on cotranslational folding, specificity of the FRET approach, action of suppressor mutations, and folding intermediates as druggable targets. In this revision the discussion has been re-written to address these salient points.

***Reviewer comment:***

*6a- The authors show (by both FRET and thermal sensitivity) that the collapse defect caused by the A455E or L558S mutation is resolved for nascent chain lengths >744 aa (Figures 2c-f, 4a, 5b). But in vivo, clearly everything does not “get better”, as these mutations lead to less mature CFTR formed. Why, precisely, do these mutant CFTR proteins fail to mature?*

**Reply:** This finding surprised us as well. While we do not fully understand the implications, we feel that this result demonstrates a fundamental complexity of de novo folding. It is obvious from our results and that of others, that in addition to the defect observed here, some folding defect remains following synthesis of the NBD1 domain that is not detectable by FRET. This is not surprising given that the FRET assay does not report on all aspects of domain structure. We speculate that apparent “resolution” of the folding defect may be due to: i) the inability of the probe sites to detect a residual folding abnormality, ii) a decrease in domain stability similar to that proposed for F508del, or iii) subtle changes that may disrupt late folding events such domain-domain assembly. These possibilities are now discussed in the revised manuscript (pages 16-17).

***Reviewer comment:***

4

Shishido H. et al.	Point by point reply to the reviewer’s comments

6b- *Why did some of the 10 disease-causing N-terminal subdomain and helical domain mutations not cause a defect in chain compaction? Is this merely due to the FRET pair used here being insensitive to the effects of these mutations (i.e., there IS an effect on collapse, but not in the dimension measured by this FRET pair)? Or are these mutations more likely altering something else about CFTR folding?*

**Reply:** We can only speculate why some mutations did not generate defects that were picked up in our assay. NBD1 folding clearly involves a complex series of coupled steps, some cotranslational, and others posttranslational. Thus, it is possible that some mutations introduce structural changes that simply do not affect the cotranslational pathway (now discussed on page 8). Alternatively, mutations may induce a structural change that was undetected by the FRET-pairs used here. It is perhaps not surprising that mutations impact more than one property of folding. For example, F508del decreases NBD1 thermal stability and also interferes with NBD1-TMD2 inter-domain interactions. The finding that mutations might exert different structural effects is also consistent with evidence that small diverse molecules designed to restore F508del folding correct an overlapping but not identical group of folding mutations. This is now mentioned in our revised manuscript (pages 16-17).

***Reviewer comment:***

6c- *Why do the PT suppressor mutations improve folding only of A455E and not L558S? Particularly if, as the MD results suggest, the two are essentially in contact with one another in the folding intermediate?*

**Reply:** Although many suppressor mutations have been described in the past two decades for F508del, the precise mechanism by which most act is still unknown. Previous studies have suggested that S492P increases rigidity of N-terminal α-helical subdomain linker^4^, and I539T increases surface hydrophilicity of the NBD1 α-helical subdomain^5^. The S492P/I539T combination was originally reported to partially correct F508del folding, although the precise mechanism of suppression at the molecular level is not well understood even for F508del^4,6^ (discussed pages 13-14). Indeed, we tested numerous other suppressor mutations that had no effect on the folding intermediates.

With regard to selective suppression of A455E, the disruptive mechanism of L558S might be in part due to its greater effect on β-core compaction (Fig. 2e,f), i.e. affecting a later stage of folding. However, introduction of a bulky negatively charged residue into the NBD1 core (A455E) versus a relatively small hydrophilic residue into the α-helical subdomain (L558S) is likely to impact NBD1 folding differently, particularly after the domain is synthesized and the overall fold is acquired, while the precise mechanism of suppression at the molecular level is not well understood for either suppressor mutations even for F508del^4,6^. This point is now discussed in the revised manuscript (pages 18-19).

5

**Shishido H. et al.	Point by point reply to the reviewer’s comments**
Reviewer #2

***Reviewer comment:***

*The manuscript by Shishido et al. describes the effect of several CF-causing mutations on the folding process of NBD1. The study used a FRET-based systems previously described and investigated by the authors. By using combination of various truncation, reporter placement and ribosome detachment, the authors identify mutations that modulate the folding process reporters and suggest that it could provide insight on the pathogenesis by several (mainly two) mutants.*

*The question is highly relevant and the experimental system is elegant. The partial recovery of both FRET signal and protein maturation when introducing reverting mutation is in support of the claim.*

*However, it is it very difficult to assess whether the findings are indeed relevant to the disease mechanism or mere fluctuation due to the in vitro system.*

*In particular, one would need some sort of negative control to evaluate whether the (moderate) changes in FRET signal observed in the CF-causing mutants are not seen in the case of non-disease causing mutation. The problem of course is that several disease-causing mutants (that prevent proper CFTR folding) do not affect the FRET pattern.*

**Reply:** This point was also made by Reviewer 1. To demonstrate that the findings are relevant to disease mechanism, we examined two additional CFTR variants M470V and G551D as negative controls. M470V is a well-known polymorphism that by itself, does not cause disease, whereas G551D is a CF-causing gating mutant that does not affect CFTR folding or trafficking. Analyses of serial NBD1 truncations containing these mutations are now provided in Supplementary Figure 2 and described on page 10. In both cases, no detectable changes in compaction (FRET) were observed at the critical stages of α-helical subdomain and β-core folding. Thus, the normal de novo folding pathway for CFTR is highly reproducible for wild-type and normally folding variants. Conversely, deviations from this pathway are indicative of certain disease-causing mutants and not simply a result of fluctuation of the assay. It is also important to note, that not all disease-causing mutations exert the same effect on protein compaction (Fig. 1). These issues are further addressed in response to Reviewer 1 and now also discussed in the revised manuscript (pages 16-17).

***Reviewer comment:***

*Of course the most striking one is the absence of change for the F508del, which is known to affect folding and stability of NBD1 and can be reverted by changes in the helical domain of NBD1. The authors have already briefly discussed this in previous publication (Kushoo 2011), speculating that F508del may affect later stages of the folding process. Considering the dramatic effects previously observed for F508del on both NBD1 and full length expression, folding and maturation in various expression systems, this remains very difficult to understand, especially since the FRET based system used here is designed to detect quite detailed changes in the folding*

**6**

Shishido H. et al.	Point by point reply to the reviewer’s comments
*process.*

**Reply:** This is an excellent point that was investigated extensively in the more recent paper by Kim et al. (Science 2015)^7^. In that study (Fig. S8), we examined a series of truncated polypeptides containing F508del in the context of R487UAG and D567UAG (acceptor sites). Consistently, FRET profiles did not reveal detectable differences from the wild-type nascent polypeptides, indicating that the F508del mutation does not influence specific folding events as monitored by these probe sites under these conditions. First, it is important to note that the crystal structure of F508del NBD1 is nearly identical to wild-type, and the trafficking defect of full-length F508del is temperature sensitive and weakly reversed by incubating cells at low temperature. In our system, translation is performed at 24°C. Second, it is also not at all clear whether the trafficking defect for F508del that is observed in cells is a direct effect of cotranslational NBD1 folding, NBD1 domain stability, defective interactions between NBD1 and TMD2, or a combination of these defects. While the FRET assay is very sensitive, detection of a given folding defect is dependent on how the distance between the two available probes sites is affected by a specific perturbation. Thus we do not expect to detect all cotranslational folding defects with the FRET assay. Rather, as with any assay system, the value lies in its ability to distinguish a clear difference between two defined polypeptides under rigorously controlled experimental conditions. While we would have liked very much to characterize the F508del defect with our assay, for reasons now discussed on pages 16-17 that was not the case. In our opinion, this does not negate the ability to detect defects induced by other mutations.

***Reviewer comment:***

*The main indication that the changes in signal observed for A455E and L558S are revealing the mechanism of pathogenesis comes from the fact that second site suppressor revert the folding defect and at the same time improves expression for A455E. However, while the FRET signal is identical to wt (suggesting no folding defect), the maturation recovery is limited to 20% (note that the broken axis in Fig6 is misleading). Obviously something else is going on, which is not related to the changes in FRET signal initially detected. A simple interpretation is that the changes of FRET signal are not correlated to the expression defect caused by the mutants. In addition, the same suppressor mutations act efficiently on F508del, although the authors suggest that the folding perturbations are very different.*

*So it seems very difficult to reconcile the two.*

**Reply:** We thank the reviewer for raising these questions, which were not addressed adequately in our original manuscript. This criticism addresses fundamental concepts of our study, and therefore warrants a rather detailed explanation on the context surrounding CFTR cotranslational folding, misfolding, and folding correction.

In this study we examine the cotranslational NBD1 folding pathway as one aspect of a complex folding process, which is, in turn, one of five domains in CFTR. Cotranslational folding is certainly not the only way that mutations impact CFTR folding or trafficking. As discussed in the introduction, F508del disrupts CFTR folding by at least 2 mechanisms: i) directly inhibiting thermal stability of NBD1^8-10^; and ii) destabilizing an intramolecular interface between NBD1 and the 4^th^ intracellular loop in TMD2^10-12^. Of the 1700 reported

7

**Shishido H. et al.	Point by point reply to the reviewer’s comments**

**CFTR variants, there are likely several hundred disease-causing mutations that decrease CFTR folding efficiency. Some affect ER trafficking and become ERAD (proteasome) substrates; others form insoluble aggregates and are cleared by autophagy^13^; some affect protein stability at the plasma membrane^14^. Thus, mutations have widely divergent effects on CFTR folding. To date, numerous small molecules have been developed to correct F508del trafficking; three are clinically approved and at least six others have been in clinical trials, with dozens more used as tool compounds. Despite more than two decades of study, it is impossible to predict a priori, which trafficking mutation will be corrected, or the extent to which it will be corrected, by a give molecule, even though essentially all molecules were initially selected to correct F508del.**

**We firmly agree with the reviewer that there are many things going on here. Most studies have attempted to define CFTR folding defects by comparing biophysical behavior of purified proteins in solution. A major conclusion of this manuscript is that in addition to disrupting structure of purified domains or full-length protein, the cotranslational folding pathway is a previously unappreciated mechanism by which disease-causing mutations can affect folding.**

**A second point is that correction of a single folding defect is not necessarily expected to completely restore CFTR trafficking. (see response to Review 1). Our results are entirely consistent with results obtained using small molecules and genetic suppression of studies of F508del^5,12,15^. For example, I539T had a dramatic effect in restoring F508del NBD1 stability as monitored by limited proteolysis, but a very weak effect on trafficking of full-length CFTR. This observation ultimately lead to the “2-hit” hypothesis wherein efficient restoration of F508del requires correction of both defects and has been confirmed by FDA approval of Trikafta. We inadequately discussed these issues in the original version and now highlight them in the revised manuscript (pages 3, 18).**

**Regarding the different effect of the PT suppressor mutations on F508del and A455E. It is well known that missense mutations induce misfolding via different mechanisms, and that suppressor mutations, like small molecules, can have pleiotropic effects. As explained to the Reviewer 1 (item #6c), it is largely unknown how the PT suppressor mutations work. Previous studies have reported that S492P increases rigidity at the interface of the N-terminal subdomain and α-helical subdomain^4^, and I539T rescues stability of F508del NBD1^5^, possibly by increasing surface hydrophilicity. Therefore, the effect of the suppressor mutations on A455E and L558S are almost certainly due to multiple effects, the full details of which are beyond the scope of this study.**

**Regarding the Y-axis of Fig. 5g (prior Fig. 6g), we were not attempting to be confusing, but prefer the broken axis, in order to focus on the effect of the PT suppressor mutations on A455E relative to A455E alone. We now clearly point out “~20% wild-type level” in the text to describe the result on page 14.**

***Reviewer comment:***

*In the end one wonders why a transient folding defect is relevant for the disease considering that extending the truncation point leads to wt-like the FRET folding pattern. Indeed, in the patient’s cell the entire polypeptide is synthesized so it is not obvious why, as stated by the authors, the*

**8**

**Shishido H. et al.	Point by point reply to the reviewer’s comments**

*folding intermediates would play such an important role in the pathogenesis, or at least the ones observed here.*

**Reply:** This question was also raised by the Reviewer 1 (item #6a), and it is difficult to answer definitively. We agree that prior to this study, it was not at all obvious that transient folding defects would be relevant for disease pathogenesis, nor indeed, that such defects would exist at all. In fact, that was the basic question we sought to address. Our previous work showed that the coordinated timing of NBD1 subdomain folding is critical for proper CFTR folding, and we also showed that altering the NBD1 translation rate with synonymous mutations (a transient event during synthesis) induced CFTR aggregation.

Thus we speculated that disease-causing mutation might also influence cotranslational folding and thereby impact the final folding outcome. Consistent with this we showed that NBD1 folding, specifically the timing of folding events, is altered by A455E and L558S. Suppressor mutations that correct this defect, also partially restore CFTR trafficking. These findings by themselves were quite novel. We also agree, as the reviewer points out, that eventual compaction of the NBD1 domain was unexpected. While we do not fully understand the implications, we speculate that this may be due in part to the high contact order of NBD1 wherein C-terminal residues provide additional stabilizing contacts to enable domain compaction as detected by FRET (now discussed on page 18). Therefore, one possibility is that these particular CF-causing mutations act by altering the normal order of NBD1 subdomain compaction. We have now mentioned this possibility in the revised manuscript (page 18). We also discuss that the spatial resolution of our FRET approach is insufficient to quantify the precise nature of the folding defect. Lack of a change in FRET yields limited information regarding other potential perturbations. And it is likely that some residual folding defect that is not detected by FRET may still lead to CFTR misfolding. We now discuss this point in the revised manuscript (pages 16-17).

***Reviewer comment:***

*Specific points:*

*-There is no clear discussion on the possible correlation (or absence of) in the type of mutation*

*studied and the FRET/folding signal. Once again, as F508del is expected to the typical folding*

*deficient mutant and nothing is observed.*

**Reply:** In this study, we examined ten CF-causing mutations known to disrupt CFTR trafficking (Class II mutations) and thus predicted to disrupt CFTR folding (Sosnay, P.R. et al., Nature Gent. 2013)^16^. A455E was also reported as a Class III^17,18^, and V mutation as the reviewer mentions below. As previously shown in our earlier study no detectable effect of F508del was observed in our FRET assay (Kim S. et al., Science 2015)^7^, indicating that the F508del mutation does not profoundly influence the folding events as monitored by the acceptor sites (R487UAG and D567UAG) used under the conditions of these experiments. This is likely due to the temperature sensitive nature of the mutation (in vitro translation is done at 24**°C**) and the nearly identical crystal structure as mentioned on pages 8, 16-17. Despite the presence of dozens (possibly hundreds) of CFTR Class II folding mutations, no clear structural information has been obtained for

**9**

**Shishido H. et al.	Point by point reply to the reviewer’s comments**

the vast majority of these, only a broad grouping within structural regions (core domains, interfacial regions, etc) of CFTR. A non-peer reviewed study by Philip J. Thomas ([**http://hdl.handle.net/2152.5/1033**](http://hdl.handle.net/2152.5/1033))) has attempted to sub-classify class II mutations based on preliminary data that A455E, S492F, I507del, F508del, A559T, R560K, and R560T are Class IIa mutations that are predicted to alter the folding of the domain, and F508del, V520F, and L558S are Class IIb mutations that are predicted to alter association of two or more domains. However, little is known regarding how they actually affect domain-domain interactions. Thus, a systematic comparison in this study is extremely difficult.

***Reviewer comment:***

*-Mutation A455E is a class V mutant, thus where CFTR is functional but produced in insufficient quantities, resulting a mild phenotype. It can be rescued by correctors C3 and C4. The authors should at least have tried those in their assay.*

**Reply:** Small molecules hold great potential both for understanding pathology of inherited protein folding disorders and for therapeutic benefit. It is difficult, however, to predict how small molecules might reverse specific abnormalities. Even a single mutation can have multiple effects on folding (as demonstrated by F508del). And many mutations likely have no cotranslational effect at all. It is well known that Lumacaftor partially corrects F508del folding and that it is also active for a subset of other Class II mutations. This is also true for Elexacaftor (unpublished data). Despite much work in this area, activity against one mutation is not predictive of others. We did not include results of testing Lumacaftor, C3, C4 and other small molecules in this study for several reasons. First, none of these molecules impacted NBD1 cotranslational folding intermediates in our hands. Second, as to whether folding intermediates could be targets for small molecule screening is a much larger question and deserving of its own study. Towards that end, we have recently developed a high-throughput screening system to optically detect and quantify FRET in nascent polypeptides immobilized on agarose beads. We have carried out a 2,000 compound pilot screen in 384 well format, and have recently completed a 50,000 compound screen in 1,536-well format that we hope to submit soon for publication.

We have tested C3 and C4 using NBD1 A455E truncated at residue 654 (acceptor site at R487UAG) with negative and show results here in below. It is not at all surprising that C3 and C4 do not work during the NBD1 synthesis^15^ because the likelihood that a given corrector would work by this precise mechanism on this precise intermediate is

extremely low, given the complexity of CFTR folding and the various ways that it can be disrupted by hundreds of misfolding mutations.

**10**

Shishido H. et al.	Point by point reply to the reviewer’s comments

| FRET | 40% 30% 20% 10%  0% |  | |
| --- | --- | --- | --- |
|  |  |  |  |

0	5	10	15

[C3] (uM)

|  | 40%  30% |  |  |  |  |  |  |
| --- | --- | --- | --- | --- | --- | --- | --- |
|  |  |  | | | |  |  |
| FRET |  |  |  |  |  |  |  |
|  | 20%  10% |  |  |  |  |  |  |
|  |  |  |  |  |  |  |  |
|  | 0% |  |  |  |  |  |  |
|  |  |  | 5 10 15  [C4] (uM) | | | | |

***Reviewer comment:***

*-In Fig1, all data show the FRET signal at truncation 654. According to Fig 2 that is where the strongest difference are seen between wt and either A455E or L558S. Thus a similar figure at different truncations may reveal a different profile. Why was this position chosen for the screening?*

**Reply:** The reviewer is correct that we might observe a different FRET profile for different truncations. Our reason for choosing truncation 654 was based on our previous results (Kim S. et al., Science 2015)^7^ that truncation represents a particularly critical folding intermediate where α-helical subdomain folding and 13-sheet core formation are temporally coupled, and highly sensitive to perturbations in translation dynamics. This is a critical intermediate for formation of the 13-sheet core, which appears to require cotranslational folding events. We have now mentioned this point in the manuscript (page 7).

***Reviewer comment:***

*-The data from in Fig3 is very difficult puzzling and not well discussed. If folding is properly achieved once reaching position 568, why extending it to 604 would perturb the folded state/intermediate? That seems to go against the idea of co-translational folding. Stating that this is due to the ribosome being around seems like a simplistic statement. Have the authors demonstrated that the ribosome does not interact with the CFP (which could affect FRET signal).*

**Reply:** We have extensively examined effects of the ribosome on CFP folding and previously published results describing cotranslational folding implications for CFP and several other fluorescent proteins. The ribosome does not affect CFP folding, maturation kinetics, or chromophore formation if the length of the tether from the CFP C-terminus to the ribosome peptidyl transfer center is more than 45 amino acids (Kelkar, D.A., et al., *J Biol Chem 2012*)^19^. That study also established several key principles, which were used here and in our previous studies that allowed us to directly compare the state of compaction for a ribosome-tethered polypeptide to an equivalent polypeptide that has been released from the ribosome. In each FRET measurement, the absolute CFP fluorescence intensity (pps/nM) is measured and shown to remain constant. This point is now covered in the discussion section (page 16).

11

**Shishido H. et al.	Point by point reply to the reviewer’s comments**

Regarding ribosome effects described in Figure 3, the text has been extensively

rewritten in the revised manuscript (pages 10-12). We agree that this data and its interpretation is complex and apologize for not explaining it clearly enough in the original manuscript. We and others have previously shown that the ribosome can exert an entropic effect on certain tethered nascent polypeptides outside the exit tunnel^7,20^. The precise nature of this effect is not yet defined, and it appears to be more prevalent for certain peptides. Our data indicates that the destabilizing effect of the ribosome acts to augment the destabilizing effect of the CF-causing mutation, which we show first becomes evident at truncation 604. Thus, ribosome-attachment augments the effect of the mutations on NBD1 folding intermediates. We cannot rule out that the possibility that molecular chaperones such as HSP40, HSP70, and HSP90, which are abundant in RRL, might also play a role in subdomain compaction. We now discuss this point on pages 17­18.

***Reviewer comment:***

*-Fig5: The determination of the thermal stability of NBD1 biosynthetic intermediates of A455E and L558S by this assay is not clear since only the absolute FRET efficiency is different (only for the intermediate state?) but the slopes for both mutants are the same as the wt-NBD1. One would expect a shift at which Efret diminution starts. This further calls for a positive control, mutant with known effect on thermal stability.*

**Reply:** We agree and have revised the text and our interpretation of the data in Fig. 4 (prior Fig. 5) to reflect this limitation (pages 12-13). Unfortunately, we do not have a positive control to test thermal stability, because the folding defect of a well-known temperature-sensitive mutant F508del is not detected by the location of our FRET probes as we mention in the result section (page 8) and in the discussion section (pages 16-17).

***Reviewer comment:***

*Minor points:*

*- p28: PDB:5UAK is not a crystal structure of NBD1.*

Now correctly refers to CFTR CryoEM structure**.**

*- There are a numerous typographical errors, for example*

*p.11:”truncat*ons”., “determine*4*d*

*P. 17 : Lumacaftor misspelled 3 times.*

*p.33: Figure 2a b-acti*c*n*

*-Fig 3 and fig4 should be grouped obviously.*

**Reply:** We apologize for any spelling errors and incorrect information of the PDB. We made a special effort to check for and correct these errors in the revised manuscript. We have also combined Fig. 3 and Fig. 4.

**12**

**Shishido H. et al.	Point by point reply to the reviewer’s comments**
Reviewer #3

***Reviewer comment:***

*The work of Shishido and colleagues uses a cell free translation system and FRET measurements to get insight into the in vivo folding process of the NBD1 domain of the CFTR protein, by analysing gross structural properties of NDB1 fragments of increasing chain length that are ribosome bound. Likewise the system simulates the in vivo co-translantional folding of this particular model protein. The methodologies applied are sound but they are not novel. However, the problem of understanding protein folding in the cell is of crucial importance. Furthermore, the model system investigated has a strong biomedical interest because CFTR is the causing agent of cystic fibrosis. These two points, together with the fact that the article is written for a broad general audience makes the work potentially interesting and appropriate for nature communications. Unfortunately, I cannot recommend its publication in its present form. The reasons are as follows.*

*1. The focus of the article is on a specific structural intermediate and how its perturbation by mutation disrupts the in vivo folding pathway. To be very honest I don’t think the authors present enough data to back-up their conclusions, at least as they suggest in the abstract and introduction of the paper. Let me explain why. In the protein folding literature a folding intermediate is a conformation populated along the folding pathway. Furthermore, a specific intermediate means that it is possible to structurally characterize it (including via NMR), and clearly specify its structural features (even with atomic detail). However, there is not in this work any piece of data backing up the idea of a specific folding intermediate. In fact the only property accessed is degree of compaction, and the authors highlight as relevant the coupling between the alpha-helical subdomain and the beta-core. Indeed, while reading the paper I noticed many instances mentioning an intermediate but the concept the is not consistent or clear. Let me give some examples:*

*Abstract – “...a specific structural intermediate...”*

**Reply:** We have removed the term from the sentence (page 2).

*Introduction (p5) – “two mutations (A455E and L558S) in different region of NBD1 disrupt compaction of a specific folding intermediate; ...”*

**Reply:** We have replaced this sentence with ” two mutations (A455E and L558S) in different regions of NBD1 delay cotranslational compaction...” to describe our results more precisely (page 5).

*Introduction (p7) – “The ability to monitor NBD1 folding while the polypeptide remains*

*attached to the ribosome is appealing because it allows access to folding intermediates that exist only transiently during CFTR synthesis. By translating a series of RNA transcripts truncated at defined lengths, it is therefore possible to capture kinetically stalled, transient translation intermediates that reflect snap shots of the folding process, and thus reconstruct the relative compaction of different subdomains as a function of nascent polypeptide length.”*

**13**

**Shishido H. et al.	Point by point reply to the reviewer’s comments**

**Reply:** Because the terms “folding intermediates” and “kinetically stalled translation intermediates” used here are appropriate descriptors in this study, we kept them here with references in the revised manuscript (page 7). To clearly define these terms and avoid confusion, we have added a more precise definition in the same paragraph as follows: “An important caveat is that truncated polypeptides may lack critical native stabilizing contacts and therefore likely represent structural ensembles rather than a predominant structural intermediate. Truncated cohorts are therefore referred to simply as nascent polypeptides or folding intermediates. Structural perturbations detected by changes in FRET thus reflect changes in the ensemble that ultimately gives rise to the final folded structure.” (page 7)

*Introduction (p7) – “Hence, a nascent polypeptide truncated at residue 654 examines the effects of mutations on folding intermediates as the fl- sheet core is still in the process of acquiring its native structure. This is a particularly critical intermediate state given that α-helical subdomain folding is temporally coupled to fl-sheet core formation and its premature folding disrupts NBD1 final structure”*

**Reply:** We have replaced these sentences with “This truncation represents a critical stage of translation in which the *13*-sheet core is in the process of acquiring its native structure^7^, which in turn is temporally coupled to *α*-helical subdomain compaction^7^. Mutations that alter the timing of α-helical subdomain compaction for this intermediate state would therefore most likely negatively impact CFTR folding.” (page7)

*Introduction (p7) – “also decreased compaction of the fl-sheet core in this folding intermediate (Fig. 1e). These results indicate that disease-causing mutations can cotranslationally perturb structural intermediates of ribosome-attached nascent polypeptides.”*

**Reply:** As the reviewer pointed out as below, we did not study “structural intermediates”. Therefore, these sentences were replaced with “decreased compaction of the *13*-sheet core (**Fig. 1e**). Thus, multiple trafficking mutations can cotranslationally perturb NBD1 folding intermediates.” (page 7)

*Results (p12) –“ We next examined the effect of the A455E and L558S on thermal stability of NBD1 intermediates by subjecting ribosome-bound nascent polypeptides to progressive thermal denaturation...”*

**Reply:** The term of “NBD1 intermediates” has been replaced with “NBD1 folding intermediates” as defined for consistency on page 7 in the revised manuscript. (page 12)

*Discussion (p14) – “transiently disrupt a specific intermediate as detected by FRET during a brief window of synthesis;”*

**Reply:** The term “a specific intermediate” has been removed and the sentence has been revised to “exert detectable effects during a brief window of synthesis that involves the coordinated folding of the α-helical subdomain and the β-sheet core”. (page 15)

**14**

**Shishido H. et al.	Point by point reply to the reviewer’s comments**

*Discussion (p14) – “The de novo protein folding landscape is characterized by sequential folding intermediates (or structural ensembles) that exist transiently and are constantly remodeled as the nascent polypeptide progressively emerges from the ribosome exit tunnel during synthesis”*

**Reply:** We have revised this sentence to describe the de novo folding landscape more appropriately as follows: “The de novo protein folding landscape is characterized by folding intermediates, likely comprised of structural ensembles, that exist transiently and are sequentially remodeled as the nascent polypeptide progressively emerges from the ribosome exit tunnel” (page 15)

*Discussion (p14) – “The current study shows that disease-related mutations disrupt physical compaction of a specific structural intermediate, thereby directing the nascent polypeptide into off-pathway events that lead to a final misfolded state”*

**Reply:** This sentence has been replaced with “our results now demonstrate that mutations can also exert their effect by disrupting folding intermediates along the folding pathway and thereby alter folding outcome”. (page 16)

*This collection of sentences shows that the authors mention specific structural intermediates, specific folding intermediates, kinetically stalled intermediates, sequential folding intermediates (or structural ensembles (?) – how can this be specific?(!)), as being the same thing. And they clearly are not. So, the first thing the authors should do is to clearly define what an intermediate is in their study. Based on the presented data and to my understanding one can only think of kinetically stalled intermediates (which do not have a specific structure and therefore represent a structural ensemble). If this is the case the conclusions of the paper are much weaker than anticipated by the authors. If this is not the case and the intermediate is specific, then the authors should present data that shows its specific structural traits. Degree of compaction is clearly not enough. I also do not understand how is it that one can conclude from the presented data that the population of the referred ‘intermediate’ is linked to a productive CFTR trafficking.*

**Reply:** We appreciate the reviewer’s comments and recognize that our imprecise language relating to a “specific structural intermediates” reflected a confusing terminology that was not as precise as it should have been. In this revision, we have followed the reviewer’s advice and have eliminated the terms “structural intermediate” and “specific structural intermediate”, and now use the more appropriate descriptors “kinetically stalled folding intermediates” or simply “folding intermediates”, which we clearly define in the revised manuscript (pages 7 and 15).

Two caveats that we would point out relate to the N-terminal subdomain, which we showed (Khushoo A. et al., Mol. Cell 2011) folds cotranslationally into a sufficiently stable structure to form a functional ATP binding site^3^. We also observed for the each probe site and each peptide region studied that the nascent polypeptide transitioned from a state of low FRET to a plateau similar to that observed for the full length domain, and in all cases, the maximal FRET value remained constant for prolonged periods of

**15**

Shishido H. et al.	Point by point reply to the reviewer’s comments

synthesis. Because Acceptor probe sites were chosen to have optimal proximity to the Donor probe in the fully folded structure, this indicates to us, a transition from a more disordered ensemble to a locally restricted ensemble similar to that of the full-length folded domain.

As the reviewer notes, the output of our study is admittedly crude with respect to NMR. However, our primary goal was not to structurally define cotranslational folding intermediates, but rather to demonstrate the impact of disease-causing mutations on such folding intermediates, which may have relevance to pharmacological correction.

Regarding the reviewer’s last comment “*I also do not understand how is it that one can conclude from the presented data that the population of the referred ‘intermediate’ is linked to a productive CFTR trafficking.*”. We agree that it is quite difficult to attribute any observed folding defect, including ones shown here, directly to its effect on CFTR trafficking. This is true for the majority of CFTR missense mutations where precise defects and the mechanisms by which they disrupt full-length CFTR folding are unknown. Traditional studies examining mechanistic effects of F508del have relied almost entirely on i) purified NBD1 protein produced in heterologous expression systems (often containing multiple stabilizing mutations) or on ii) cis-acting genetic complementation approaches. As a result of such studies, purified recombinant CFTR domains have become valuable tools for drug discovery. To date, there have been no studies investigating whether any folding mutations have an impact on CFTR folding intermediates. We feel that this is an obvious and relevant question for three reasons. NBD1 folding is intrinsically complex; the efficiency of NBD1 folding is a limiting step in CFTR biosynthesis^12,21,22^, and NBD1 does not reversibly fold in vitro^8,9^.

Moreover, our previous studies showed that NBD1 folding involves critical

cotranslational events that are required for efficient CFTR folding and processing^7^. The fundamental principle addressed here is whether CF-causing trafficking mutations might also impact features of cotranslational folding intermediates. We feel that our results address this question directly. If the reviewer is asking whether the specific defect(s) we have identified are fully responsible for the mis-trafficking of mutant CFTR, the answer is no. We can no more reach that conclusion than we can definitively say what portion of the thermal instability observed for purified recombinant F508del NBD1 domain is directly responsible mis-trafficking of F508del CFTR. However, in addition to demonstrating an effect on cotranslational folding, we have shown that genetic complementation of the cotranslational defect partially restores trafficking of full-length CFTR in cells using similar genetic approaches and to a very similar extent as initial “suppressor mutations” for F508del (Fig. 5).

Taken together these findings demonstrate that trafficking mutations can disrupt the cotranslational folding landscape and as such, may be targets for small molecule drugs as has been proposed for the FDA approved drug Lumacaftor. We propose that these folding intermediates may play a role in CFTR trafficking but again, the precise mechanisms by which any CFTR mutation impacts overall maturation are still unknown^21^. Whether these or other intermediates are actually druggable entities, will require further study. Towards that end we have developed a high throughput screening assay to test this hypothesis. We now cover these issues in the discussion section (pages 18-19).

16

Shishido H. et al.	Point by point reply to the reviewer’s comments

***Reviewer comment:***

2. *Part of the presented data is based on molecular simulations. As stated in the article the simulations should provide insight into the nature of the transient folding defect (presumably this is the perturbation of degree of compaction). Could the authors specify the intramolecular potential used in their model? Also, could the authors clearly explain the reason why they explore the folding dynamics of NBD1 fragment truncated between residues 389-354? I don’t see why this fragment should provide insight into the experimental data, especially because the experimental data is based on several truncated fragments that are tethered to the ribosome, and the simulations are carried out in bulk conditions. Since tethering clearly constraints the conformational space I don’t see how one can compare the two scenarios (simulations and in vitro), or use the simulations to get insight into structural details taking place during the in vitro folding. It would be much adequate if the simulations could be carried out for one or more fragments of the WT protein tethered to the ribosome, and then for the same fragments with the two selected mutations (i.e. A455E and L558S). A direct comparison of the two model systems could provide more insight on the hypothesis placed by the authors that “these mutations may destabilize a partially collapsed hydrophobic interface that is transiently present during NBD1 translation.” As it stands I don’t see why the simulations are useful. They provide a prediction but the prediction was not tested. On the other hand, since the tethering scenario was not considered, the experimental data can not be interpreted based on the simulations.*

**Reply:** We completely agree. After multiple discussions with our collaborators, and after expending significant effort to address the reviewer’s suggestions directly, we reached the conclusion that a thorough study involving simulations of i) NBD1 polypeptides truncated at multiple residues, ii) tethered and untethered to the ribosome, iii) for wild-type, and multiple mutations would require significant resources beyond the scope of this study. While we found the results intriguing as presented in the original manuscript, we agree that they were insufficiently robust to reach meaningful conclusions. We therefore felt that the suggested studies would best be completed in the format of an independent computational study. We have therefore removed the molecular simulation data from this revised manuscript.

***Reviewer comment:***

3. *Other points:*

*What exactly is the rationale for focusing the study on the A455E and L558S mutations? This is*

*not clear.*

**Reply:** We apologize for any confusion. All of the mutations shown in Figure 1d&e were chosen based on work from the Thomas lab that systematically examined NBD1 mutations for their effect on trafficking^16^, Due to the tedious nature of these experiments we were forced to limit the number of mutations that we could study in more depth. From the initial screen, we identified three mutations, A455E, L558S, and A559T, which demonstrated statistically significant folding disruption of both α-helical subdomain and

17

Shishido H. et al.	Point by point reply to the reviewer’s comments

β-sheet core. Since residue L558S is adjacent to residue A559T, and L558S shows a more severe folding disruption based on our FRET data (Fig. 1d&e), we focused on two mutations that were located within N-terminal and α-helical subdomains. We acknowledge that subsequent work on other mutations may yield additional insight into this area. Importantly, our work does not imply that these are the only mutations that disrupt cotranslational folding, nor that the defects we have identified reflect a universal pathway. We have now revised the manuscript to show the rationale why we have focused on the A455E and L558S mutations (page 8).

***Reviewer comment:***

*There is a recent study (DOI: 10.1039/c6mb00193a) where it is predicted via molecular simulations that substation of residue 492 would restore the folding of F508del-NBD1 and, concomitantly, the surface expression and/or function of F508del-CFTR. This study should be mentioned.*

**Reply:** We thank the reviewer for the suggestion. We have now mentioned this study and cite the reference (reference #56) in the revised manuscript (page 13).

**References**

1. Matsumura, Y., David, L.L. & Skach, W.R. Role of Hsc70 binding cycle in CFTR folding and endoplasmic reticulum-associated degradation. *Mol Biol Cell* **22**, 2797-809 (2011).
2. Carlson, E., Bays, N., David, L. & Skach, W.R. Reticulocyte lysate as a model system to study endoplasmic reticulum membrane protein degradation. *Methods Mol Biol* **301**, 185-205 (2005).
3. Khushoo, A., Yang, Z., Johnson, A.E. & Skach, W.R. Ligand-driven vectorial folding of ribosome-bound human CFTR NBD1. *Mol Cell* **41**, 682-92 (2011).
4. Estacio, S.G., Martiniano, H.F. & Faisca, P.F. Thermal unfolding simulations of NBD1 domain variants reveal structural motifs associated with the impaired folding of F508del-CFTR. *Mol Biosyst* **12**, 2834-48 (2016).
5. Hoelen, H. et al. The primary folding defect and rescue of DeltaF508 CFTR emerge during translation of the mutant domain. *PLoS One* **5**, e15458 (2010).
6. Aleksandrov, A.A. et al. Allosteric modulation balances thermodynamic stability and restores function of DeltaF508 CFTR. *J Mol Biol* **419**, 41-60 (2012).
7. Kim, S.J. et al. Protein folding. Translational tuning optimizes nascent protein folding in cells. *Science* **348**, 444-8 (2015).
8. Protasevich, I. et al. Thermal unfolding studies show the disease causing F508del mutation in CFTR thermodynamically destabilizes nucleotide-binding domain 1. *Protein Sci* **19**, 1917-31 (2010).
9. Wang, C. et al. Integrated biophysical studies implicate partial unfolding of NBD1 of CFTR in the molecular pathogenesis of F508del cystic fibrosis. *Protein Sci* **19**, 1932-47 (2010).
10. Thibodeau, P.H. et al. The cystic fibrosis-causing mutation deltaF508 affects multiple steps in cystic fibrosis transmembrane conductance regulator biogenesis. *J Biol Chem* **285**, 35825-35 (2010).
11. Rabeh, W.M. et al. Correction of both NBD1 energetics and domain interface is required to restore DeltaF508 CFTR folding and function. *Cell* **148**, 150-63 (2012).

18

Shishido H. et al.	Point by point reply to the reviewer’s comments

12. Mendoza, J.L. et al. Requirements for efficient correction of DeltaF508 CFTR revealed by analyses of evolved sequences. *Cell* **148**, 164-74 (2012).
13. Houck, S.A. & Cyr, D.M. Mechanisms for quality control of misfolded transmembrane proteins. *Biochim Biophys Acta* **1818**, 1108-14 (2012).
14. Okiyoneda, T. et al. Peripheral protein quality control removes unfolded CFTR from the plasma membrane. *Science* **329**, 805-10 (2010).
15. Okiyoneda, T. et al. Mechanism-based corrector combination restores DeltaF508-CFTR folding and function. *Nat Chem Biol* **9**, 444-54 (2013).
16. Sosnay, P.R. et al. Defining the disease liability of variants in the cystic fibrosis transmembrane conductance regulator gene. *Nat Genet* **45**, 1160-7 (2013).
17. Van Goor, F., Yu, H., Burton, B. & Hoffman, B.J. Effect of ivacaftor on CFTR forms with missense mutations associated with defects in protein processing or function. *J Cyst Fibros* **13**, 29-36 (2014).
18. Veit, G. et al. From CFTR biology toward combinatorial pharmacotherapy: expanded classification of cystic fibrosis mutations. *Mol Biol Cell* **27**, 424-33 (2016).
19. Kelkar, D.A., Khushoo, A., Yang, Z. & Skach, W.R. Kinetic analysis of ribosome-bound fluorescent proteins reveals an early, stable, cotranslational folding intermediate. *J Biol Chem* **287**, 2568-78 (2012).
20. Kaiser, C.M., Goldman, D.H., Chodera, J.D., Tinoco, I., Jr. & Bustamante, C. The ribosome modulates nascent protein folding. *Science* **334**, 1723-7 (2011).
21. Kim, S.J. & Skach, W.R. Mechanisms of CFTR Folding at the Endoplasmic Reticulum. *Front Pharmacol* **3**, 201 (2012).
22. Mendoza, J.L. & Thomas, P.J. Building an understanding of cystic fibrosis on the foundation of ABC transporter structures. *J Bioenerg Biomembr* **39**, 499-505 (2007).

**Reviewer comments, second version:**

Reviewer #1 (Remarks to the Author):

Shishido et al. are to be commended for their thorough and detailed response to reviewer concerns. I wholeheartedly recommend this article for publication. I do, however, suggest the authors include in their introduction and discussion three very new studies co-translational folding (below) that strengthen the relevance of their work. Each underscores Shishido et al.'s points regarding the complexity of folding mechanisms for large proteins and the potential for the non-equilibrium co-translational folding process to positively impact folding yield in ways that are not obvious from merely gazing at the native protein structure:
-co-translational folding of complex, multi-domain proteins (https://doi.org/10.1016/j.molcel.2019.01.043)
-co-translational folding as a strategy to avoid kinetic traps on complex energy landscapes (https://doi.org/10.1073/pnas.1913207117)
-the impact of translation (and elongation rate) on folding of a complex multimeric protein (https://doi.org/10.1073/pnas.1907126117)


Reviewer #2 (Remarks to the Author):

After a long period, the authors have returned a revised version of the manuscript were a number of questions raised initially have been addressed.

There are however, several outstanding issues that, in my opinion, have not been resolved. Specifically, the central issue is whether the slight changes of E-FRET are relevant to the pathogenesis.

1/ F508del
The fact the most important mutation, F508del, which is known to affect folding is not changing the FRET signal remains very puzzling. . It has been shown/proposed that F508del misfolding happens co-translationally (see work by the Braakman lab). In contrast the authors suggest that F508del may not be affecting the folding (as measured by the FRET assay) because it is temperature sensitive and the experiment is performed at 24°C. A simple way to address that would be to change the temperature. While this may affect translation, controls may be able to identify key events. Furthermore the authors have performed thermal stability study on nascent polypeptides for their target mutation (A455E and L558S). Also, claiming that “crystal structure of F508del NBD1 is nearly identical to wild-type” is misleading as all structures bearing F508del also include stabilizing mutations.

2/ Intermediates
That fact that mutations only affect truncated mutant and not full domain folding is not properly addressed by the authors in their reply. They claim that it is “difficult to answer” and that’s about it. In the full length protein, the entire polypeptide is synthesized and the mutations have dramatic effects. The fact that nothing is seen my FRET if the full NBD1 domain synthesized in vitro suggest that the assay is not suited for this. Note that A455E mutation is absolutely extreme from a structural standpoint. A455 is a small aliphatic residues buried in interface of the A/B subdomain. Changing it to a Glu is expected to have dramatic consequence. That fact that the entire NBD1 seems to fold fine in the presence of the mutation in the FRET assay further suggests that the assay does not detect relevant changes.

3/ Compaction
The mutations discussed here change the FRET signal from about 37% to 27% (from what I could see on Fig1). I performed a simple calculation from a predicted R0 of 38.5 Angstroms (according to the original paper by the authors). The FRET change indicates a distance change of less then 4 angstroms only Can we really infer “compaction” difference with such a minute distance change ? This is also difficult to reconcile with the dramatic biological effect of the mutations.

The experimental system of the authors is elegant, but in my opinion the present data does not convincingly show that it can dissect the effect of mutations on folding.

On a different theme, I am taken aback by the showcasing of the Vertex drugs in the introduction. This is not relevant for the present study (especially since they did not try these drugs in their assay).


Reviewer #3 (Remarks to the Author):

The authors have substantially changed the manuscript. I feel that the conclusions are now backed up by the experimental data and the text is overall more accurate. Unfortunately the authors decided not to carry on the simulation work that could provide mechanistic insight into their experimental findings and strengthen their results. This is a week point of the revised version. However, I feel that the reported results are still important and may be published in Nature Communications.

**Author rebuttal, second version:**

***Reviewer #1 (Remarks to the Author):***

*Shishido et al. are to be commended for their thorough and detailed response to reviewer concerns. I wholeheartedly recommend this article for publication. I do, however, suggest the authors include in their introduction and discussion three very new studies co-translational folding (below) that strengthen the relevance of their work. Each underscores Shishido et al.'s points regarding the complexity of folding mechanisms for large proteins and the potential for the non-equilibrium co-translational folding process to positively impact folding yield in ways that are not obvious from merely gazing at the native protein structure:*

*-co-translational folding of complex, multi-domain proteins*
[*(*](https://nam03.safelinks.protection.outlook.com/?url=https%3A%2F%2Fdoi.org%2F10.1016%2Fj.molcel.2019.01.043&data=01%7C01%7Cwskach%40cff.org%7C8d1952b22f75470a53a008d7c72782c4%7Cf581bf53aa87446b9a4fd85c3510df63%7C0&sdata=R502jMVjTd8S60EM5h26cNAXKcJ%2Bn6vcZ5s%2FrDdQSQc%3D&reserved=0)*https://doi.org/10.1016/j.molcel.2019.01.043)*

*-co-translational folding as a strategy to avoid kinetic traps on complex energy landscapes* [*(*](https://nam03.safelinks.protection.outlook.com/?url=https%3A%2F%2Fdoi.org%2F10.1073%2Fpnas.1913207117&data=01%7C01%7Cwskach%40cff.org%7C8d1952b22f75470a53a008d7c72782c4%7Cf581bf53aa87446b9a4fd85c3510df63%7C0&sdata=78xtm3KUIy7GIFZZQprtVv0sUG0NN8Csa%2BwI1%2Fmslgw%3D&reserved=0)*https://doi.org/10.1073/pnas.1913207117)*

*-the impact of translation (and elongation rate) on folding of a complex multimeric protein* [*(*](https://nam03.safelinks.protection.outlook.com/?url=https%3A%2F%2Fdoi.org%2F10.1073%2Fpnas.1907126117&data=01%7C01%7Cwskach%40cff.org%7C8d1952b22f75470a53a008d7c72782c4%7Cf581bf53aa87446b9a4fd85c3510df63%7C0&sdata=%2FBcTapv4oyY6P0kfB0hCK9K%2B%2Ffrg4R0rwRiR8YNUF2E%3D&reserved=0)*https://doi.org/10.1073/pnas.1907126117)*

**Reply**: We thank the reviewer for pointing out these references that we had inadvertently overlooked. The three studies are now cited and discussed in the revised manuscript on pages 4, 15-16, and 18 (Ref# 37, 45, and 67).

***Reviewer #2 (Remarks to the Author):***

*After a long period, the authors have returned a revised version of the manuscript were a number of questions raised initially have been addressed.*

*There are however, several outstanding issues that, in my opinion, have not been resolved. Specifically, the central issue is whether the slight changes of E-FRET are relevant to the pathogenesis.*

***1/ F508del***

*The fact the most important mutation, F508del, which is known to affect folding is not changing the FRET signal remains very puzzling. . It has been shown/proposed that F508del misfolding happens co-translationally (see work by the Braakman lab). In contrast the authors suggest that F508del may not be affecting the folding (as measured by the FRET assay) because it is temperature sensitive and the experiment is performed at 24°C. A simple way to address that*

**Shishido H. et al.	Point by point reply to the reviewer’s comments (2^nd^ round)**

*would be to change the temperature. While this may affect translation, controls may be able to identify key events. Furthermore the authors have performed thermal stability study on nascent pol****ypeptides for their target mutation (A455E and L558S). Also, claiming that “crystal structure*** *of F508del NBD1 is nearly identical to wild-****type” is misleading as all structures bearing*** *F508del also include stabilizing mutations.*

**Reply:** We previously proposed three possible explanations as to why F508del, had no detectable effect on FRET measurements in our assay system (Kim, et al. ^1^). The first possibility is that while F508del impacts CFTR folding outcome, it may not actually impact NBD1 translation intermediates. Second, subtle alterations in NBD1 folding intermediates might not be detected by the specific probe sites used here to measure FRET. Third, potential effects might be masked by the reduced temperature of translation (24°C) which suppresses the folding defect in cells (also discussed in the current revision on page 8). The reviewer**’s concerns**, which focus on the first and the third possibilities are discussed below.

We agree with the Reviewer that the elegant pulse labeling experiments from the Braakman lab provide evidence for increased protease susceptibility of F508del NBD1 relative to wild-type NBD1 during CFTR synthesis (Hoelen, et al. ^2^). However, these studies examined full length CFTR (containing TMD1, NBD1, R domain, TMD2 and NBD2), in a cell expression system where it is difficult to synchronize translation. The study was therefore not designed to determine precisely when misfolding occurred because the identify of polypeptides giving rise to NBD1 proteolytic fragments could not be determined. Given that the F508del mutation causes at least two folding defects: i) a direct decrease in NBD1 thermal stability; and b) destabilization of the interface between NBD1 and the 4^th^ intracellular loop in TMD2^3,4^, the Braakman data are equally consistent with either a direct cotranslational effect on NBD1, or an indirect effect later during synthesis caused by disruption of domain-domain contacts due to the absence of Phe508. We are unaware of any direct experimental evidence that F508del cotranslationally perturbs NBD1 folding and respectfully feel that the Braakman data, with regard to F508del, do not completely negate the validity of our assay.

For the third possibility, technical limitations prevented us from preparing ribosome-nascent chain complexes (RNCs) after translation at 37°C. RNC production for FRET requires three events to take place, i) translation of the peptide, ii) incorporation of the acceptor probe at the engineered UAG stop codon by the modified suppressor tRNA, and iii) a stable peptidyl-tRNA bond that allows isolation of intact RNCs. As shown below, increasing the translation temperature to 37°C had a relatively modest effect on overall translation efficiency as suggested by the reviewer (terminated peptide+readthrough peptide+peptidyl-tRNA). However, there was a marked (>95%) decrease in stop codon readthrough, and stability of the peptidyl-tRNA bond at 37°C such that intact peptidyl-tRNA was essentially undetectable. Given that NBD1 constructs typically yield only 1-5nM of purified RNCs under optimal translation conditions, it is simply not possible to generate enough RNCs to measure FRET following translation at 37°C. Results for 30°C translation are shown for completeness, but F508del misfolding in cells is also suppressed at this temperature.

Shishido H. et al.	Point by point reply to the reviewer’s comments (2^nd^ round)

**Figure 1. Increasing translation temperature to 37°C decreases stop codon read-through efficiency and peptidyl-tRNA stability.** (**A**) Representative autoradiograms (SDS-PAGE) of in vitro-translated ^35^S-labeled CFP-NBD1 (truncation 744) containing the R487UAG codon translated at 24, 30 or 37°C in the presence of Lys-tRNA^amb^. Exogenous bovine tRNA was titrated at 40 or 120 ng/µl as described in Methods to optimize translation efficiency. Arrows indicate location of polypeptides that either terminate at the engineered UAG, read-through the UAG codon but lack tRNA, or retain the peptidyl-tRNA bond and therefore derive from intact RNCs. (**B**) Graphs show quantitation of band intensity of total translated protein (terminated + readthrough polypeptides + peptidyl-tRNA), readthrough protein (readthrough polypeptide + peptidyl-tRNA) and peptidyl-tRNA as indicated at the top of the graphs. Note that band intensities are normalized in each graph to the amount of product recovered under our standard translation conditions (24°C and 40 ng/µl tRNA). Peptidyl-tRNA detected at 37°C in presence of 40 or 120 ng/µl the tRNA was 0.01 and 0.00, respectively.

We thank the review for the comment related to NBD1 crystal structures, and now acknowledge the presence of stabilizing mutations in F508del NBD1 studies on page 17.

***2/ Intermediates***

*That fact that mutations only affect truncated mutant and not full domain folding is not properly addressed by the authors in their reply. They claim that it is “difficult to answer” and that’s about it. In the full length protein, the entire polypeptide is synthesized and the mutations have dramatic effects. The fact that nothing is seen my FRET if the full NBD1 domain synthesized in vitro suggest that the assay is not suited for this. Note that A455E mutation is absolutely extreme from a structural standpoint. A455 is a small aliphatic residues buried in interface of the A/B subdomain. Changing it to a Glu is expected to have dramatic consequence. That fact that the entire NBD1 seems to fold fine in the presence of the mutation in the FRET assay further suggests that the assay does not detect relevant changes.*

**Reply**: We apologize that our previous response was deemed insufficient and agree that high-resolution structural data showing the effect of the A455E mutation would be valuable. Unfortunately no such data has yet been published, so the actual structure of the mutant remains unknown. This observation is discussed on page 17. In the revised manuscript (Page 18) we fully acknowledge that the apparent compaction of NBD1 at the completion of synthesis is unexpected. However, in the absence of data we do not see how this observation negates the clear differences observed in FRET at shorter chain lengths. We speculate that at short chain lengths, there are a limited number of residues to stabilize the folding intermediate, and distal C-terminal residues might provide additional stabilizing energy that impacts the effective distance between FRET

**Shishido H. et al.	Point by point reply to the reviewer’s comments (2^nd^ round)**

probes. Such a process seems reasonable in a domain with high contact order such as NBD1. However, we also agree with the Reviewer that, *“...despite the apparent compaction of the domain as measured by FRET (****Fig. 2c-f****), some residual folding defect must still remain in the full-length CFTR protein as demonstrated by its poor processing efficiency (****Fig. 2a****).”*. Given the complex multidomain structure of CFTR, our data would suggest that the precise nature of this folding defect is not simple. As the Reviewer has pointed out, PT suppressor mutations, which restore the in vitro cotranslational defect at short NBD1 chain lengths, only partially restore folding of the full-length protein in cells. Thus, in a manner analogous to F508del, cotranslational misfolding of A455E likely results in a local perturbation in NBD1 that is undetected by FRET, which impacts a subsequent alteration (e.g. domain-domain interactions) in the full-length protein. We now discuss these possibilities more clearly on pages 18-19 in the revised manuscript.

Regarding the nature of the A455E mutation, we agree that a structural change in NBD1 would be expected for reasons outlined by the Reviewer. In our discussion, we provide several possibilities that might explain our results (pages 16-18). However, structural studies that could provide insight for or against these arguments are not available as noted above. In the absence of structural data, we have done our best to be transparent about the limitations of our system, and to interpret the data in light the considerable literature on known and predicted folding features of CFTR. As stated above, we do not see how the absence of a change in FRET at one truncation, should be used to invalidate clear evidence for a folding defect at a different truncation based on a theoretical consideration. The relevance and impact of our findings come from the following observations: i) there are clear effects on folding intermediates induced by disease-causing mutations, which delay compaction of specific regions of NBD1, ii) manifestations of these folding defects are directly and reproducibly influenced by translation machinery including the ribosome, iii) genetic suppression of the cotranslational defect improves overall folding efficiency of the intact protein in cells.

We agree, however, and have discussed at some length that our assay is not perfect, and like any scientific assay, has certain limitations that need to be considered in drawing conclusions. Thus we are not proposing that the FRET assay can prove the absence of a defect in the full-length domain, only that we were unable to detect one, and in fact fully acknowledge that some residual defect does in deed exist despite our findings.

***3/ Compaction***

*The mutations discussed here change the FRET signal from about 37% to 27% (from what I could see on Fig1). I performed a simple calculation from a predicted R0 of 38.5 Angstroms (according to the original paper by the authors). The FRET change indicates a distance change of less then 4 angstroms only Can we really infer “compaction” difference with such a minute distance change ? This is also difficult to reconcile with the dramatic biological effect of the mutations.*

*The experimental system of the authors is elegant, but in my opinion the present data does not convincingly show that it can dissect the effect of mutations on folding.*

**Shishido H. et al.	Point by point reply to the reviewer’s comments (2^nd^ round)**

**Reply**: We have previously reported gradual increases in FRET efficiency for multiple wild type NBD1 subdomains as the nascent polypeptide grew in length^1,5^ and provided evidence that these changes reflect on-pathway folding events that correspond to acquisition of a native ligand (ATP) binding site. Examination of wild-type NBD1 and two naturally occurring mutations that do not impact folding (M470V and G551D) support our experimental trajectory for the normal folding pathway (Supplemental Figure 2).

Detailed analysis of the two mutations studied here (A455E and L558S) show a reproducible and unequivocal deviation from this pathway. From these data, we concluded that the FRET system can detect nascent polypeptide transitions from an elongated (unfolded), conformation to a compacted (folded) conformation, and thus, the potential ability to reflect a conformational change in the nascent polypeptides caused by the mutations.

We agree with the Reviewer that it is theoretically possible to calculate the distance between D and A fluorophores based on FRET efficiency. However, this type of distance calculation is only meaningful in a rigid system where the two probes are held at a fixed distance relative to one another. We respectfully take issue that actual distances can be inferred in a structural ensemble of nascent folding intermediates without deep knowledge of the ensemble characteristics, which is not possible here. Other than validation of FRET measurements by varying simple tether lengths in developing the FRET assay (Khushboo et al^5^), we have specifically avoided these sorts of calculations, recognizing that they would likely be immediately challenged on theoretical grounds.

Predicting the magnitude of FRET change between two donor and acceptor probe sites for a specific perturbation (single aa change) of a nascent tertiary folding intermediate in such a system is therefore quite difficult. It is important to consider that measurements here, cannot be used to infer how distances might or might not change in other regions of the nascent polypeptide. Finally, we have defined “compaction” operationally based on the well accepted distance-dependence of FRET and have deliberately avoided over-extrapolating changes in FRET to specific structural features. Rather, we have only attempted to interpret changes in FRET efficiency that were associated with carefully control changes in nascent chain length and probe sites, with or without the CF-causing mutations, while all other biological parameters were held constant.

*On a different theme, I am taken aback by the showcasing of the Vertex drugs in the*

*introduction. This is not relevant for the present study (especially since they did not try these drugs in their assay).*

**Reply:** We thank the reviewer for this comment. It was not our intention to showcase the Vertex drugs so much as to highlight recent advances in pharmacologically improving CFTR folding and its impact on peoples lives. We agree that some of information is not directly relevant to our study and removed some of this language while retaining some important points related to folding intermediates (page 3).

***Reviewer #3 (Remarks to the Author):***

*The authors have substantially changed the manuscript. I feel that the conclusions are now backed up by the experimental data and the text is overall more accurate. Unfortunately the authors decided not to*

Shishido H. et al.	Point by point reply to the reviewer’s comments (2^nd^ round)

*carry on the simulation work that could provide mechanistic insight into their experimental findings and strengthen their results. This is a week point of the revised version. However, I feel that the reported results are still important and may be published in Nature Communications.*

**Reply**: We appreciate the reviewer**’s comments and recommendation**. We agree that the simulation data would have potential strengths in the original manuscript, and are also disappointed that we were not able to include the simulation work for reasons described in the previous point-by-point reply.

**References**

1. Kim, S.J. et al. Protein folding. Translational tuning optimizes nascent protein folding in cells. *Science* **348**, 444-8 (2015).
2. Hoelen, H. et al. The primary folding defect and rescue of DeltaF508 CFTR emerge during translation of the mutant domain. *PLoS One* **5**, e15458 (2010).
3. Mendoza, J.L. et al. Requirements for efficient correction of DeltaF508 CFTR revealed by analyses of evolved sequences. *Cell* **148**, 164-74 (2012).
4. Rabeh, W.M. et al. Correction of both NBD1 energetics and domain interface is required to restore DeltaF508 CFTR folding and function. *Cell* **148**, 150-63 (2012).
5. Khushoo, A., Yang, Z., Johnson, A.E. & Skach, W.R. Ligand-driven vectorial folding of ribosome-bound human CFTR NBD1. *Mol Cell* **41**, 682-92 (2011).

**Reviewer comments, third version:**

Reviewer #2 (Remarks to the Author):

First, I want to stress that I find the experimental system developed by the authors elegant and interesting. It allows to probe with fine resolution intrinsic folding behavior.

That being said, the main point of contention remains the interpretation of the data.
In a sense, it seems that the authors are only keen on interpreting positive results but discard negative data as simple limitations of the system.

Coming back to the Braakman studies, the main conclusion on these in cellulo studies of full length CFTR folding was “the primary folding defect arises during synthesis, as soon as NBD1 is translated.” Sure, differences in the systems may explain differences with the data discussed here, and -surely- the authors may be right when stating that “the Braakman data, with regard to F508del, do not completely negate the validity of our assay.” But I would say that the cellular system may be more relevant than the in vitro translation that lacks a number of key molecular actors.
Furthermore; the effect of F508del on NBD1 folding is also supported by the data from the same group in their 2005 Molecular Cell paper (Kleizen et al) where they used in vitro translation as well.

Note that if, as suggested by the authors “alterations in NBD1 folding intermediates might not be detected by the specific probe sites used here to measure FRET” it could also be that the wt NBD1 isn’t entirely well folded in this assay. The authors previously demonstrated that the in-vitro-translated NBD1 can bind ATP which suggest proper folding. However, NBD1 is made of different subdomains that can unfold separately and ATP binding happens in a different subdomain than the alpha subdomain where F508 is located.

The fact that the difference in FRET signal is only observed in a limited synthesis window (and not when translating the entire NBD1) surely indicates that different folding pathways may be at play in the different mutants (and thus different intermediates). But it also suggests that the end result is the same in all cases, i.e. that independently of the pathways, NBD1 is well folded in all of the variants.

The authors make the point that maybe the FRET data does not pick differences in folding state of the entire domain (“Thus we are not proposing that the FRET assay can prove the absence of a defect in the full-length domain, only that we were unable to detect one,”)>

So if I follow that reasoning, the idea if that when the system detects a change it is meaningful, and when it does not, well there could still be changes, we just don’t detect them. That feels like wishful thinking.

In my opinion, in the absence of corroborating data, the interpretation of the authors overreaching. The authors conclude that
“Taken together, our results indicate that cotranslational folding intermediates play an important role in i) the underlying mechanisms of de novo protein folding, ii) the molecular pathology of protein folding disorders, and iii) potential approaches for correcting defects caused by trafficking mutations. “

While I agree with 1, I would strongly disagree with 2 and 3. The authors have identified differences in the folding pathways, but they have not identified folding disorders in the mutants as measured by compaction of the entire domain.

I maintain that the study is elegant and reveal interesting behavior. I do also think that the interpretation of its physiopathological relevance must be tuned down.

**Author rebuttal, third version:**

***Reviewer #2 (Remarks to the Author):*** *First, I want to stress that I find the experimental system developed by the authors elegant and interesting. It allows to probe with fine resolution intrinsic folding behavior.

That being said, the main point of contention remains the interpretation of the data.
In a sense, it seems that the authors are only keen on interpreting positive results but discard negative data as simple limitations of the system.

Coming back to the Braakman studies, the main conclusion on these in cellulo studies of full length CFTR folding was “the primary folding defect arises during synthesis, as soon as NBD1 is translated.” Sure, differences in the systems may explain differences with the data discussed here, and -surely- the authors may be right when stating that “the Braakman data, with regard to F508del, do not completely negate the validity of our assay.” But I would say that the cellular system may be more relevant than the in vitro translation that lacks a number of key molecular actors.
Furthermore; the effect of F508del on NBD1 folding is also supported by the data from the same group in their 2005 Molecular Cell paper (Kleizen et al) where they used in vitro translation as well.*  *Note that if, as suggested by the authors “alterations in NBD1 folding intermediates might not be detected by the specific probe sites used here to measure FRET” it could also be that the wt NBD1 isn’t entirely well folded in this assay. The authors previously demonstrated that the in-vitro-translated NBD1 can bind ATP which suggest proper folding. However, NBD1 is made of different subdomains that can unfold separately and ATP binding happens in a different subdomain than the alpha subdomain where F508 is located.*

**Reply**: We appreciate the reviewer’s arguments and cannot rule out the possibility that WT NBD1 may not be completely folded in RRL. We now acknowledge this point on Page 17 and state: “It is also possible that in this system, the isolated WT NBD1 domain does not reach a completely folded native structure, thereby allowing FRET values to converge at longer chain lengths.”

*The fact that the difference in FRET signal is only observed in a limited synthesis window (and not when translating the entire NBD1) surely indicates that different folding pathways may be at play in the different mutants (and thus different intermediates). But it also suggests that the end result is the same in all cases, i.e. that independently of the pathways, NBD1 is well folded in all of the variants.*

**Reply**: See comment above.

*The authors make the point that maybe the FRET data does not pick differences in folding state of the entire domain (“Thus we are not proposing that the FRET assay can prove the absence of a defect in the full-length domain, only that we were unable to detect one,”)>

So if I follow that reasoning, the idea if that when the system detects a change it is meaningful, and when it does not, well there could still be changes, we just don’t detect them. That feels like wishful thinking.*

**Reply**: We have acknowledged that the assay used here has certain limitations discuss these limitations at some length on pages 18-19 to allow the readers to follow our logic in drawing conclusions. With regard to the issue of A455E mutant folding, we now state more transparently (page 19) that: “…cotranslational misfolding of A455E likely results in a local perturbation in NBD1…” .

*In my opinion, in the absence of corroborating data, the interpretation of the authors overreaching. The authors conclude that
“Taken together, our results indicate that cotranslational folding intermediates play an important role in i) the underlying mechanisms of de novo protein folding, ii) the molecular pathology of protein folding disorders, and iii) potential approaches for correcting defects caused by trafficking mutations. “

While I agree with 1, I would strongly disagree with 2 and 3. The authors have identified differences in the folding pathways, but they have not identified folding disorders in the mutants as measured by compaction of the entire domain.

I maintain that the study is elegant and reveal interesting behavior. I do also think that the interpretation of its physiopathological relev*ance must be tuned down.

**Reply**: We agree with reviewer’s concerns, and in response, have amended these conclusions, in several locations of the discussion as follows.

1) As noted above, we now acknowledge that we have not unequivocally established that WT NBD1 is fully folded in our system and that this may account for FRET results at longer truncations (Page 17).
2) In response to the reviewers objection, we have toned down Point 2 to state that our results on cotranslational folding intermediates “raise the possibility that they contribute to the molecular pathology…” (Page 20)
3) We have deleted Point 3 from the final paragraph of the discussion and simply acknowledge that further studies are needed to determine whether cotranslational folding intermediates might be druggable targets. (Page 20)
